# Integrating single-nucleus barcoding with spatial transcriptomics via Stamp-seq to reveal immunotherapy response-enhancing functional modules in NSCLC

**DOI:** 10.1038/s41421-025-00861-6

**Published:** 2026-02-05

**Authors:** Yitong Pan, Huan Yan, Jinhuan Han, Rui Wu, Caiming Xu, Guang Lei, Xingyong Ma, Ying Guan, Zhao Li, Junyuan Deng, Keyu Li, Qingquan Wei, Guangxin Zhang, Lei Liu, Ajay Goel, Zhou Yang, Shaozhuo Jiao, Yongchang Zhang, Chenxi Tian

**Affiliations:** 1 Computation Biology Department, China National Center for Bioinformation, Beijing, China; 2 Beijing Institute of Genomics, Chinese Academy of Sciences, Beijing, China; 3 Furong Laboratory, Central South University, Changsha, Hunan China; 4 Early Clinical Trial Center, Hunan Cancer Hospital/The Affiliated Cancer Hospital of Xiangya School of Medicine, Central South University, Changsha, Hunan China; 5 School of Clinical Medicine, Qinghai University, Xining, Qinghai China; 6 SeekGene BioSciences Co. Ltd., Beijing, China; 7 Department of Molecular Diagnostics and Experimental Therapeutics, Beckman Research Institute of City of Hope, Biomedical Research Center, Monrovia, CA USA; 8 Department of Radiation Oncology, Hunan Cancer Hospital and the Affiliated Cancer Hospital of Xiangya School of Medicine, Central South University, Changsha, Hunan China; 9 GeneMind Biosciences Company Limited, Shenzhen, Guangdong China; 10 Department of Endoscopy, Shanghai Tenth Hospital, Tongji University, Shanghai, China; 11 City of Hope Comprehensive Cancer Center, Duarte, CA USA; 12 Department of Medical Oncology, Shanghai East Hospital, Tongji University School of Medicine, Shanghai, China; 13 Department of Oncology, Shanghai Medical College, Fudan University, Shanghai, China

**Keywords:** Bioinformatics, Non-small-cell lung cancer, Cancer microenvironment, Cancer immunotherapy

## Abstract

Deciphering the spatial organization of cell states is fundamental for understanding development, tissue homeostasis and disease. Emerging advances in spatial transcriptomic profiling techniques allow transcript localization but face limitations in unambiguous cell state assignments due to cellular boundary inference, low gene detection and prohibitive cost. Here, a method, Stamp-seq, is developed that leverages custom-fabricated high-density DNA sequencing chips to label single nuclei with restriction enzyme-cleavable spatial barcodes. Stamp-seq spatial barcodes are distributed at a density of 1.6 μm on the chip, allowing for single physical cell resolution with precise subtype classification and spatial mapping (with an average 4 μm localization error) and reduced cost. We utilize Stamp-seq to delineate chemoimmunotherapy-responsive cellular ecosystems in non-small cell lung carcinoma, including a distinct *IGHG1*^*+*^ plasma cell-enriched community. Through a novel application of Stamp-seq to spatially resolve BCR clonotypes, we elucidate the spatiotemporal trajectory of treatment-potentiating *IGHG1*^*+*^ plasma cells, which originate from tertiary lymphoid structures (TLSs) or the vasculature, migrate through antigen-presenting CAF (apCAF)-enriched survival niches, and ultimately contact tumor cells. We highlight the power of spatial cellular subtyping and molecular tracking using Stamp-seq and suggest that the *IGHG1*^*+*^ plasma cell niche is a better prognostic biomarker for the chemoimmunotherapy response.

## Introduction

Understanding the cellular organization and interactions within native microenvironments is fundamental to unraveling the complex biological processes involved in development, physiology, and disease, with direct implications for advancing clinical diagnostics and precision therapies. Spatial multiomics technologies have emerged as critical tools in this pursuit, enabling the integration of molecular profiles with their spatial context in intact tissues. An ideal spatial technology would have high performance, which includes high-resolution molecular detection, precise molecular localization and the ability to bin molecules at the true single-cell level without contamination from neighboring cells, as well as practical processing timelines and cost-effectiveness. However, current approaches fail to simultaneously fulfill all these criteria. The first category of methods is imaging-based methods^1,2^, which achieve high-resolution in situ transcript detection but are constrained by limited gene detection throughput under practical experimental time spans and inferred cell boundary segmentation. Next-generation sequencing (NGS)-based methods^3–5^, on the other hand, offer cost-effective, genome-wide profiling at subcellular resolution but are confounded by inevitable transcript mixing resulting from the horizontal diffusion and vertical joining of transcripts from overlapping cellular layers and inferred cellular segmentation, leading to ambiguous spatial transcript and cell assignments. Both types of strategies struggle to resolve heterogeneous and especially underrepresented cell subtypes with high fidelity, resulting in an unsatisfactory analysis of the cellular organization and interactions^6,7^. Recent innovations, such as XYZ-seq^8^ and Slide-tags^9^, address these limitations by employing spatial barcoding to achieve true single-cell resolution. However, these methods are hampered by the low spatial resolution of barcodes and the prohibitive costs associated with the sequencing of hundreds of thousands of nuclei per tissue section. Addressing these challenges is essential to unlock scalable, high-precision spatial transcriptomics for broad biomedical applications.

Here, we present Stamp-seq (single-nucleus tagging with movable and spatially resolving barcodes for high-throughput sequencing), a spatial transcriptomics method that repurposes second-generation sequencing platforms to create a chip with cleavable spatial barcodes (1.6 μm density) for precise nuclear labeling and a reduced chip fabrication cost. Spatially encoded nuclei undergo multiplex barcoding during in-tube reverse transcription, followed by high-throughput processing via droplet microfluidics, significantly increasing efficiency and reducing library construction costs. This integrated Stamp-seq method enables cost-effective total RNA analysis with spatial resolution, rapid processing, and broad tissue compatibility, specifically enabling spatially resolved cell subtype identification with a localization fidelity of an approximately half-cell radius.

Cancer immunotherapies provide significant clinical benefits for some but not all patients. An unmet need lies in identifying predictive biomarkers for stratification and potential therapy-boosting strategies. Technologies such as single-cell transcriptomics have been applied to study immune cell dynamics in multiple types of cancers during immunotherapy treatments^10–12^. However, treatment response-related spatial communities have not been explored at the single-cell level, and current studies have focused only on a panel of selected markers^13–15^.

We applied Stamp-seq to samples from patients treated with chemoimmunotherapy and identified two spatial cell communities enriched in patients with a superb response, namely, tertiary lymphoid structures (TLSs) and a plasma cell-concentrating niche outside of TLSs. Further spatial profiling of BCR clonotypes revealed the spatiotemporal recruitment of antitumor *IGHG1*^*+*^ plasma cells from TLSs or the vasculature by antigen-presenting CAFs (apCAFs), a CAF subtype capable of immune activation and antigen presentation^16^, prior to contact with tumor cells. We developed the nucleus-tagging spatial technology Stamp-seq and elucidated the spatial organization of the human antitumor immune response.

## Results

### Overview of Stamp-seq technology

**Fig. 1:**
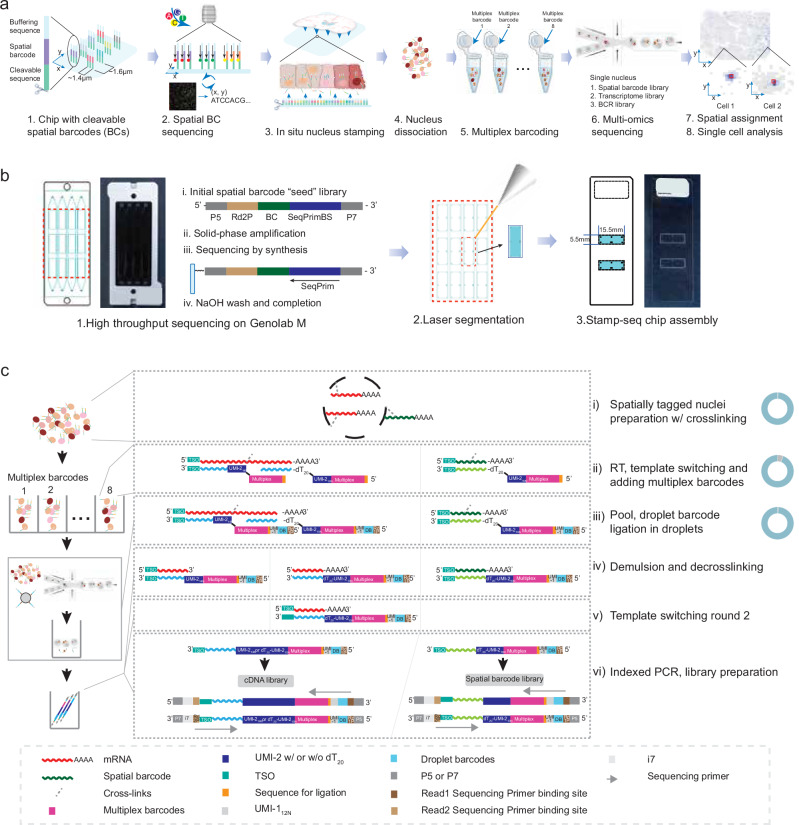
Schematic overview of the Stamp-seq workflow and chip generation. **a** Stamp-seq workflow. 1. Chip design: A Stamp-seq chip featuring cleavable spatial barcodes is designed and fabricated to enable spatially resolved single-nucleus analysis. 2. Spatial barcode sequencing: The nucleotide sequences and spatial positions of the cleavable barcodes are precisely sequenced and mapped. 3. In situ barcode delivery: Fresh-frozen tissue sections are overlaid onto the chip, facilitating the entry of cleavable spatial barcodes into cells and their subsequent binding to nuclei through perforated plasma membranes. 4. Nucleus isolation: Spatially barcoded nuclei are isolated. 5. Sample multiplexing: Isolated nuclei are partitioned into eight tubes, each labeled with a unique multiplex barcode to enable multiplexed processing. 6. Multiomics library sequencing: Comprehensive multiomics libraries are generated, incorporating spatial barcodes, single-nucleus RNA sequencing (snRNA-seq), and B-cell receptor (BCR) libraries. 7. Spatial coordinate mapping: The spatial coordinates of individual nuclei are reconstructed by integrating spatial, multiplexing, and droplet barcode distributions. 8. Data analysis: High-resolution snRNA-seq data are analyzed to derive spatially resolved transcriptomic and immune repertoire profiles. **b** Stamp-seq chip generation workflow. 1. Barcode amplification and sequencing: Spatial barcodes on the chip are amplified and sequenced using the GenoLab M sequencing platform to ensure accuracy and reproducibility. 2. Chip sectioning and assembly: The chip is laser cut into sections, and two sections are affixed to a glass slide to create a functional assay-ready device. **c** Schematic of snRNA-seq and spatial barcode library construction. The workflow involves the following steps. 1. Crosslinking spatially tagged nuclei. The pie chart illustrates the spatial barcode adhesion efficiency, where blue represents the proportion of reads with spatial barcodes (~46.5%) and gray represents the proportion of reads without spatial barcodes (~53.5%). 2. Partitioning into eight tubes for multiplex RT labeling. The pie chart above shows the multiplex barcode adhesion efficiency using the reads with spatial barcodes from step 3, where blue represents the proportion of reads with multiplex barcodes (~94.4%) and gray represents the proportion of reads without multiplex barcodes (~5.6%). 3. Droplet-based barcode ligation in an oil emulsion. The pie chart above shows the cell barcode adhesion efficiency using the reads with both spatial barcodes and multiplex barcodes from step 5, where blue represents the proportion of reads with cell barcodes (~99.2%) and gray represents the proportion of reads without cell barcodes (~0.8%). 4. Demulsification and decrosslinking. 5. Template switching replication. 6. Indexed PCR amplification and library preparation. Note that the cDNA library includes sequences from mRNAs (~59.3%), lncRNAs (~3.1%), rRNAs (~28.4%), mitochondrial RNAs (~6.96%) and others (~2.31%) in WTH1092 because of the randomized primers used in the RT reaction in step II.

Stamp-seq is performed in two sequential stages: array-seq and nuclei-seq (Fig. 1a). During the array-seq stage, a physical chip is generated, consisting of spatially barcoded RNA-capture molecules, with each barcode sequence linked to a corresponding spatial coordinate on the chip. The nuclei-seq is then applied to tissue sections placed on top of the spatial chip and uses several sequential steps, including enzymatic tagging, nuclear extraction, split-labeling, library construction using a microfluidic device and high-throughput sequencing.

The array-seq process is initiated through the solid-phase amplification of a single-stranded synthetic oligonucleotide library on the GenoLab M sequencing platform (Fig. 1b, step 1). Each “seed” oligonucleotide consists of PCR adapter sequences, out of which P5 incorporates a uracil for USER enzyme cleavage at a later step and a spatial barcode incorporating 32 randomized nucleotides. Amplification on a lawn surface coated with PCR adapters results in the generation of numerous clusters, each originating from a single “seed” molecule (Supplementary Fig. S1a). These clusters comprise oligonucleotides that are identical clones of the original seed. The spatial barcode sequences and corresponding spatial coordinates of each cluster are determined during the sequencing-by-synthesis process (Supplementary Fig. S1a). Washing with NaOH facilitates the creation of a spatially barcoded array (Supplementary Fig. S1a). Finally, laser segmentation cleaves the array into 5.5 mm × 15.5 mm regions, which are then adhered onto a glass slide to complete the Stamp-seq chip assembly (Fig. 1b, steps 2 and 3). The Stamp-seq chip shows consistent cluster morphology and density across various regions (Supplementary Fig. S1b). The mean distance between fluorescence cluster centroids is 1.64 μm, and the mean diameter of the fluorescence clusters is 1.35 μm (Supplementary Fig. S1c). The duplication rate of the cluster in each Stamp-seq region is approximately 0.03%, indicating sufficient variety for nucleus labeling (Supplementary Fig. S1d).

In the nuclei-seq step, tissue sections measuring 14–20 μm are placed on the Stamp-seq chip (Supplementary Fig. 1a, c). The spatial barcode fragments are then enzymatically cleaved and released into cells to label the nuclei in situ (Fig. 1a). The labeled nuclei are dissociated and transferred into eight individual tubes, each containing unique multiplex barcodes for further labeling (Methods; Fig. 1a). These nuclei, now labeled with both unique spatial and variable multiplex barcodes, are pooled and subjected to droplet-based encapsulation, where they undergo an additional round of labeling within the droplets. The addition of multiplex barcodes and pooling increases cell loading in the microfluidic system, resulting in an increased number of captured nuclei per run with very limited evidence of multiplet formation (Methods; Supplementary Fig. S2a, b). Subsequent library construction and sequencing reveal the spatial, sample and droplet barcodes, and cDNA sequences are acquired to generate a single-nucleus spatial gene expression matrix (Fig. 1a, c).

### Stamp-seq reveals the cellular architecture in the mouse brain

We conducted Stamp-seq on 14 μm coronal sections of the adult mouse brain encompassing the hippocampus, a region characterized by its highly stereotyped architecture, rendering it an ideal model for the validation of the effectiveness of our spatial technique^4^.

**Fig. 2:**
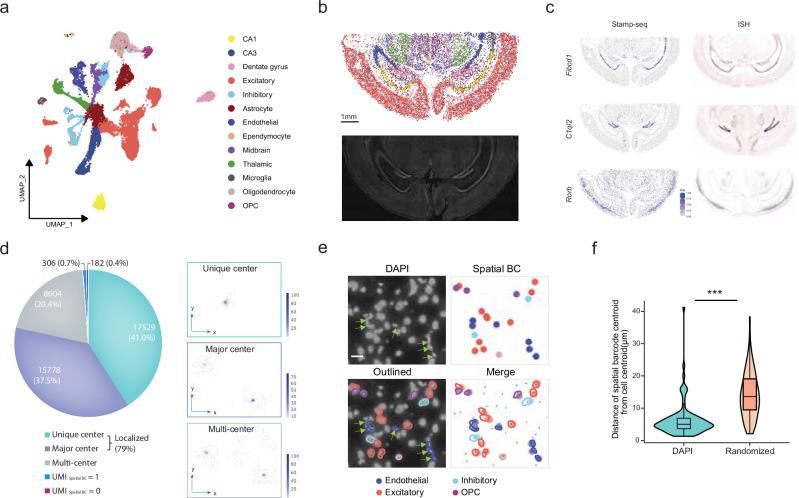
Stamp-seq enables single-nucleus spatial transcriptomics in the mouse brain. **a** UMAP embedding of snRNA-seq profiles (WTH1092), with cells color-coded by annotated cell types. CA1 Cornu Ammonis area 1, CA3 Cornu Ammonis area 3, OPCs oligodendrocyte precursor cells. **b** Stamp-seq enables high-resolution spatial mapping of nuclei in the adult mouse brain, with cells color-coded according to the same rule as in **a**. The lower image displays the “on-chip” DAPI-stained tissue section, providing a morphological reference for the sequenced region. **c** Spatial expression patterns of marker genes identified by Stamp-seq are compared against in situ hybridization (ISH) data from the Allen Mouse Brain Atlas^66^. Color scales represent normalized expression levels. **d** Spatial barcode distribution patterns that define nuclear centers in the adult mouse brain sample WTH1092. Each bin represents a 100 × 100-pixel region. The enlarged plots (right panel) depict three representative distribution patterns of nuclear centers, with the color intensity indicating the density of spatial barcode UMIs within each bin. **e** Stamp-seq-captured cells are overlaid onto a DAPI image, showing the alignment of nuclear morphology (green arrows) with cell type annotations (endothelial cells). The upper left panel highlights the nuclear shapes on the DAPI image, while the upper right panel depicts the spatial distribution and cell types of all the captured cells. The lower left panel outlines the nearest nuclei on the DAPI image, color-coded by cell type based on the Stamp-seq data. The lower right panel integrates Stamp-seq nuclear locations, cell type annotations, and the nearest nuclear outlines from the DAPI image. The black dots denote the CellProfiler-extracted centroid positions of all the nuclei. **f** Spatial distances between nuclear positions determined using Stamp-seq and their nearest counterparts identified by CellProfiler on the DAPI image are quantified. As a control, distances from randomly generated nucleus positions to their nearest DAPI-identified nuclei are compared (right panel). **e** illustrates part of the nuclei quantified in **f**. *** denotes *P* < 0.001 (Wilcoxon test).

From a coronal tissue section of 42 mm^2^, we isolated and sequenced 42,449 nuclei, of which 32,833 (77.3% of the profiled nuclei, with a median of 738 Unique molecular identifiers (UMIs) per nucleus) were localized. The resulting data were then clustered using a standard single-cell pipeline^17^ (Fig. 2a) and annotated using well-established cell type markers (Supplementary Fig. S3a and Table S1). Notably, the spatial distribution of individual cell clusters followed the anticipated pattern: astrocytes and endothelial cells exhibited a dispersed arrangement, whereas neurons of diverse subtypes maintained the characteristic laminated architecture, as evidenced by a comparison with on-chip DAPI imaging (Fig. 2b and Supplementary Fig. S3b). We further leveraged our spatially resolved Stamp-seq RNA profiles to characterize the laminar distribution of gene expression in excitatory neurons across cortical layers. This analysis revealed distinct layer-specific expression patterns of evolutionarily conserved markers, including *Cux4*, *Rorb*, and *Foxp2* (Supplementary Fig. S3c, d). Notably, the spatial expression profiles of individual genes closely matched the corresponding in situ hybridization data from reference databases (Fig. 2c).

Our spatially resolved single-nucleus localization framework comprises two sequential computational phases. Phase 1 is the identification of nuclear spatial domains through a differential analysis of Unique molecular identifier (UMI) density distributions. We computationally distinguished nuclei with unique primary and secondary spatial domains by evaluating the ratio of central versus subcentral binned spatial barcode UMI densities (Methods; Supplementary Fig. S2c). Phase 2 is coordinating optimization through minimal-distance spatial mapping. Nuclei were assigned to their most likely spatial coordinates by minimizing the combined Euclidean distance between all spatial barcode UMI distributions within their primary spatial domain and candidate pixel positions.

We employed two approaches to quantify the accuracy of spatial positioning. Initially, we calculated the spatial tag diffusion distance, defined as the distance between the assigned position and the location of all detected spatial tags for each nucleus, and discovered that the diffusion distances predominantly fell within 17 μm (50%: < 10 μm; 75%: < 17 μm; 95%: < 36 μm), confirming limited spatial tag diffusion (Methods; Supplementary Fig. S2c). We subsequently evaluated the spatial assignment accuracy of both the cell type and location. We utilized CellProfiler to extract the spatial positions of the nuclei from the on-chip DAPI image, which were defined as the “true” locations. We then placed our Stamp-seq-captured nuclei onto the on-chip DAPI image to determine the location assigned by Stamp-seq and extracted the distance between the two locations (Methods). The discrepancy in spatial alignment between the ground-truth positions and Stamp-seq-assigned locations demonstrated remarkable precision, with a mean offset of 4 μm (Fig. 2e, f and Supplementary Fig. S2d, e; Table S2). This result stands in stark contrast to simulated nuclei with randomized localizations, which exhibited a significantly larger positioning error (mean offset: 12 μm) independent of cell type considerations (Fig. 2e, f and Supplementary Table S2).

Beyond positional deviation metrics, we prioritized evaluating cell-type localization accuracy. We focused on endothelial cells, a cell class whose nuclei exhibit distinct elongated morphologies that align linearly to form vascular structures, to validate the subtype-specific spatial mapping. Stamp-seq-identified endothelial cells exhibited strong concordance with anatomical expectations: all mapped endothelial cell nuclei resided on top of or adjacent to morphologically elongated nuclei, with a subset (3/6) displaying characteristic linear alignment patterns consistent with the vascular organization (Fig. 2e).

### Stamp-seq benchmarks with other spatial transcriptomics methods in the mouse brain

We performed a systematic quality assessment comparing Stamp-seq with conventional snRNA-seq workflows to evaluate potential technical artifacts introduced by spatial barcoding. Using a rostral coronal section (42 mm²) from an adult mouse brain (Methods; Supplementary Fig. S4a, b), which is a well-characterized benchmark tissue for spatial omics technologies^3,18^, we generated Stamp-seq profiles of 17,900 nuclei with median sequencing depths of 913 UMIs and 539 detected genes per nucleus. Through reference-based integration with matched standard snRNA-seq data^19^ and our spatial datasets, we stratified cells into eight transcriptionally distinct types using canonical marker genes (Supplementary Table S3 and Fig. S4a). We found that the cell type proportions (Spearman’s *r* = 0.787; *P* = 0.02), gene expression per cell (Spearman’s *r* = 0.961; *P* < 0.001), and UMIs (Spearman’s *r* = 0.961; *P* < 0.001) were unaffected by the spatial tagging procedure (Supplementary Fig. S4b).

We conducted a systematic comparison with other state-of-the-art spatial technologies (including HDST^20^, Slide-seqV2^18^, Seq-Scope^5^, Pixel-Seq^21^, Visium-HD, Slide-tags^9^, Stereo-seq^4^, and DBiT-seq^3^) to rigorously evaluate the performance of Stamp-seq. Stamp-seq achieved high UMI and gene detection sensitivity compared with most other techniques and lower sensitivity than Slide-tags (Supplementary Fig. S4c). We further compared Stamp-seq with representative methods, such as Slide-tags, Stereo-seq and DBiT-seq, as well as public snRNA-seq data^19^ from the adult mouse brain. Uniform manifold approximation and projection (UMAP) plots and marker gene expression in cell clusters segregated using Stamp-seq revealed the clear segregation of cell clusters (Supplementary Fig. S4d–h) and specific expression of marker genes (Supplementary Fig. S4i–m). Our analysis indicates that current high-resolution platforms face critical limitations in cross-subtype transcriptome aggregation from bead/pixel-based systems, which impair clustering accuracy and marker specificity, challenges that have been effectively addressed by nuclear tagging-based spatial transcriptomic approaches such as Stamp-seq and Slide-tags.

The pairwise differential cell-type-specific marker coexpression analysis was used to compare the levels of marker coexpression in all detected or segmented cells (Methods, Supplementary Fig. S4n–r). Our results demonstrated that Stamp-seq achieved significantly higher concordance in marker coexpression patterns with public snRNA-seq data (*P* < 0.001, Wilcoxon signed-rank test) than the other spatial methods (Supplementary Fig. S4s). This quantitative alignment with gold-standard snRNA-seq profiles validates the technical superiority of our spatially resolved transcriptomic mapping, particularly in achieving reduced cross-contamination during cellular transcript capture and enabling the refined discrimination of cellular subpopulations.

### Stamp-seq identifies chemoimmunotherapy response-related cells and cellular communities

Although immunotherapy has revolutionized cancer treatment, a substantial number of patients exhibit persistent nonresponsiveness to immunotherapy^22^. Immune cell neighborhoods critically regulate immune cell recruitment/activation/exhaustion dynamics that dictate therapeutic outcomes. Single-cell sequencing delineates only changes in cell subtypes, and traditional spatial transcriptomics (imaging/NGS-based) fail to precisely define tumor immune microenvironments because of indistinct cellular segmentation boundaries. This methodological constraint underscores the discovery of therapy-relevant immune niches. We therefore performed Stamp-seq analysis of human non-small cell lung cancer (NSCLC) tumor sections obtained from patients who had undergone neoadjuvant treatment.

**Fig. 3:**
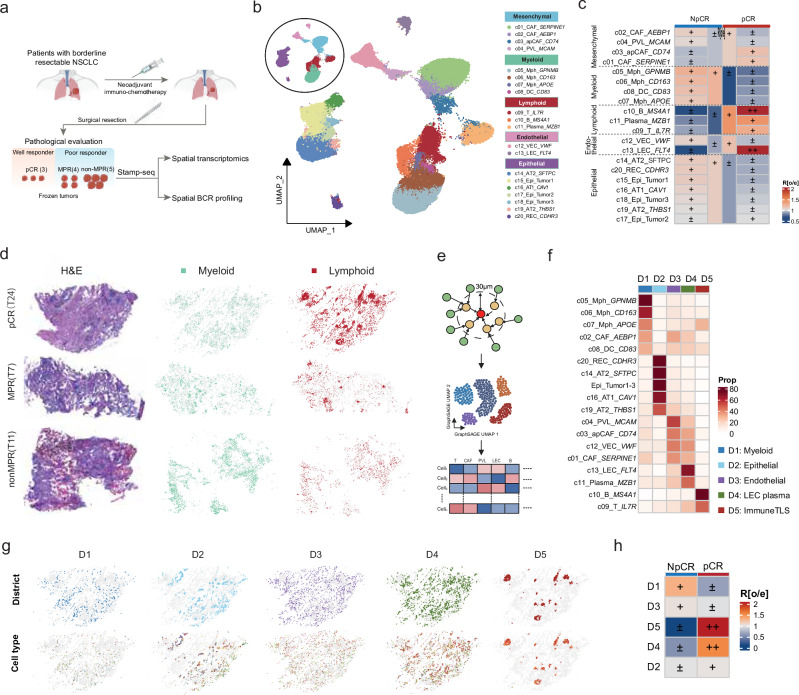
Stamp-seq enables a physical single-cell spatial analysis in non-small cell lung cancer (NSCLC) patients. **a** Schematic representation of the sample collection workflow and sequencing methodology used in this study. **b** UMAP visualization of the identified cell subsets across all analyzed cells (*n* = 125,558). **c** Heatmap displaying the tissue preference of major and minor cell subsets, which was quantified using the Ro/e score. **d** Spatial map of myeloid and lymphoid cell distribution patterns in representative samples with distinct treatment responses. **e** Graphical representation of cell communities detected using GraphSAGE. The central cell is defined based on the proportions of neighboring cell types within a 30 μm radius and clustered into five distinct cellular communities, termed “districts”. Cells without neighbors within the 30 μm radius were excluded from the analysis. **f** Heatmap showing the relative proportions of each cell type across different districts, with row normalization applied. **g** Spatial distribution of cell types (bottom panel) within each district (top panel) in the T24 pCR sample. **h** Heatmap depicting the sample preference of each district, quantified by the Ro/e score.

We obtained 12 surgically resected specimens from patients who received 2–3 cycles of neoadjuvant PD-1 blockade plus chemotherapy, which were stratified by therapeutic response as follows: 3 patients who achieved a pathological complete response (pCR), 4 patients who achieved a major pathological response (MPR), and 5 nonresponder (non-MPR) patients (Fig. 3a and Supplementary Table S4). We performed Stamp-seq to simultaneously profile transcriptomes and spatial information across approximately 120,000 nuclei (median of 551 UMIs and 438 genes per nucleus; Supplementary Fig. S5a, b). The clustering analysis of nuclei across all samples revealed distinct cell populations, including mesenchymal, myeloid, lymphoid, endothelial, and epithelial cells (Fig. 3b and Supplementary Fig. S5c). Epithelial subgroups were further classified into putative malignant tumor clusters and normal epithelial clusters based on copy number variation (CNV) scoring and the expression of *KRT9* (Supplementary Fig. S5c–e). Interestingly, we identified two transcriptionally similar AT2 subpopulations (c14 and c19; Supplementary Fig. S5c). The pseudotime analysis placed c19 earlier and c14 later in differentiation, with AT1 cells (c16) terminally differentiated (Supplementary Fig. S5f, g), which aligns with the previous finding that AT2 cells can transdifferentiate into AT1 cells^23,24^. A comparative analysis of cellular abundance between patients who achieved a pCR and non-pCR groups (NpCR, combining the MPR and non-MPR groups) revealed the significant enrichment of lymphoid cells (T cells, B cells, and plasma cells) and lymphatic endothelial cells in the pCR group, whereas myeloid cells and epithelial cells were predominantly enriched in the NpCR groups (Fig. 3c, d and Supplementary Figs. S5h and S6a, b). Notably, the c17 tumor cell subpopulation was specifically enriched in the pCR samples (Fig. 3c and Supplementary Figs. S6 and S7a). The Hallmark pathway analysis indicated that compared with NpCR tumors, residual tumor cells in pCR samples exhibited reduced activity in proliferation-related pathways (e.g., MTORC1 signaling, the G2M checkpoint, and MYC targets) but elevated TNFα and interferon α signaling (Supplementary Fig. S7b, c). Although potential sampling bias due to regional heterogeneity cannot be excluded, these findings suggest that post-treatment residual tumor cells in pCR samples (e.g., the c17 subpopulation) may activate antitumor programs to suppress proliferation. This observation is consistent with previous scRNA-seq studies reporting residual tumor cells in patients who achieved a pCR and their association with tumor recurrence^12,25^.

We implemented GraphSAGE for neighborhood-based sampling and feature aggregation of gene expression profiles from cells within a 30 μm radius, followed by unsupervised clustering of these spatial features to delineate distinct cellular niches and characterize treatment response-associated cellular communities (Methods; Fig. 3e). We identified five spatially segregated communities (D1–D5; Fig. 3f and Supplementary Fig. S8a): D1 as a myeloid-enriched niche, D2 as an epithelial cell zone, D3 as a vascular endothelial compartment, D4 as a plasma cell and lymphoid endothelial cell hub, and D5 as a T/B-cell-rich region displaying TLS features on hematoxylin and eosin (H&E)-stained sections (T24 in Fig. 3d), which we classified as an immune-TLS (Fig. 3f and Supplementary Fig. S8a). This spatial partitioning was further supported by the cellular proximity analysis, in which epithelial subgroups exhibited cohesive clustering patterns, T cells colocalized with B cells, and plasma cells were neighbors to (apCAFs; Supplementary Fig. S8b). The quantification of the spatial distribution confirmed that D3 and D4 exhibited overlapping, dispersed patterns, whereas the TLS-associated D5 niche showed maximal spatial segregation from the other compartments (Fig. 3g and Supplementary Fig. S8c). The comparative analysis revealed the pronounced enrichment of lymphoid-infiltrated niches (D4/D5) in the pCR group, whereas myeloid-dominated D1 niches predominated in the NpCR groups (Fig. 3h). These findings corroborate prior evidence linking robust T-cell infiltration and TLS formation to favorable immunotherapy outcomes, whereas myeloid cell accumulation is associated with therapeutic resistance^10,12,26^.

### Stamp-seq identifies a chemoimmunotherapy response-related fibroblastic environment

Fibroblasts, which are highly plastic and phenotypically heterogeneous stromal cells, serve as pivotal microenvironmental components that regulate immunological responses through direct cell–cell interactions. Current spatial transcriptomic methods face dual technical constraints in fibroblast characterization: suboptimal dissociation efficiency in scRNA-seq and segmentation challenges for elongated morphologies in both NGS and imaging platforms. Leveraging nuclear compartmentalization advantages, Stamp-seq achieved comprehensive fibroblast profiling (32,968 nuclei, 26.2% cellular prevalence) with unprecedented subtype classification at single-nucleus resolution.

**Fig. 4:**
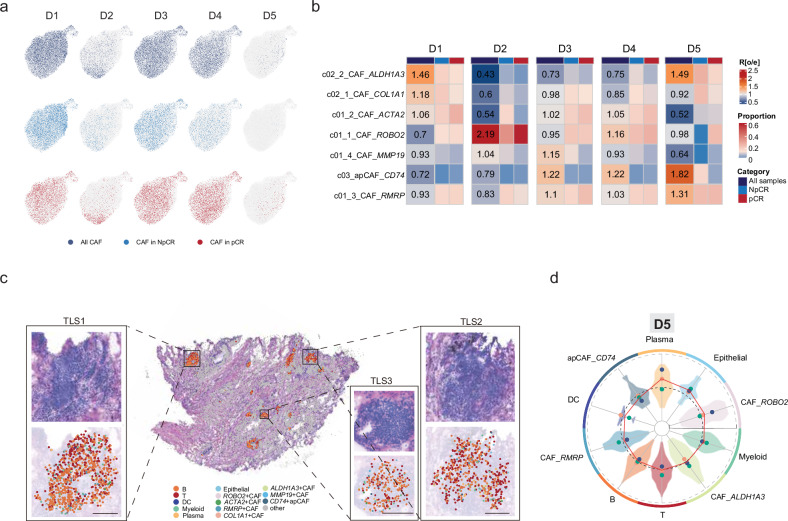
Stamp-seq revealed a chemoimmunotherapy response-related fibroblastic environment. **a** UMAP visualization of the fibroblast distribution across all samples, stratified by NpCR and pCR groups. **b** Heatmap depicting the district distribution preference of reclustered fibroblast subsets across all samples, quantified by the Ro/e score. The relative abundance of each cancer-associated fibroblast (CAF) subset within districts is shown for patients with distinct therapeutic responses (pCR and NpCR), with column normalization applied. **c** Spatial mapping of snRNA-seq profiles in sample D5, annotated by cell type, alongside the corresponding H&E-stained section of the profiled region. **d** Radar plot displaying the average distance of each cell type from the tertiary lymphoid structure (TLS) core in sample D5 of the T24 cohort. Three highly clustered TLS regions within the T24 sample were analyzed (Supplementary Fig. S8b). Embedded violin plots illustrate the radial distribution of all cells within each cell subtype.

A comparative analysis of fibroblasts across the five spatial districts (D1–D5) between the pCR and NpCR groups revealed heterogeneous UMAP distribution patterns, with pronounced disparities observed in the epithelial cell-enriched D2 and TLS-associated D5 districts (Fig. 4a). We performed a subclustering analysis to resolve fibroblast heterogeneity and identified seven functionally distinct fibroblast subtypes (Supplementary Fig. S9a, b and Table S5) and mapped their spatial enrichment patterns across treatment response groups (Fig. 4b). Notably, compared with the control samples, the pCR samples exhibited the preferential accumulation of *ROBO2*^*+*^ CAFs within the epithelial D2 niche, whereas *ACTA2*^+^/*COL1A1*^+^ myofibroblasts (myCAFs) were predominantly enriched in NpCR samples. This reciprocal distribution aligns with the reported tumor-suppressive roles of *ROBO2*^+^ CAFs, which non-cell-autonomously inhibit myCAF activation and correlate with an improved pancreatic cancer prognosis^27^. Conversely, myCAFs have been implicated in the establishment of immunosuppressive tumor microenvironments (TMEs) in early-stage NSCLC through the physical exclusion of CD8^+^ T cells and the suppression of immune cell infiltration^15^.

We further dissected the compartmentalized architecture of CAFs within TLS-containing D5 districts by quantifying the radial positioning relative to the TLS centroid in the pCR samples (Fig. 4c, d). Spatial mapping revealed that apCAFs localized to TLS cores, whereas *RMRP*^+^ and *ALDH1A3*^+^ CAFs occupied paracortical zones adjacent to T/B-cell clusters. *ROBO2*^+^ CAFs predominantly resided at TLS peripheries and colocalized with plasma cells, whereas *ACTA2*^+^/*COL1A1*^+^ myCAFs were absent. This stratified organization suggests functional specialization: apCAFs may initiate TLS formation via lymphoid chemokine secretion, *RMRP*^+^/*ALDH1A3*^+^ CAFs likely support lymphocyte differentiation, and *ROBO2*^+^ CAFs facilitate plasma cell migration out of TLSs. The exclusion of myCAFs from TLS structures further underscores their role in immune evasion, which is consistent with their enrichment in NpCR microenvironments.

### Stamp-seq identifies treatment-promoted spatiotemporal changes in the plasma cell trajectory and plasma cell niche

**Fig. 5:**
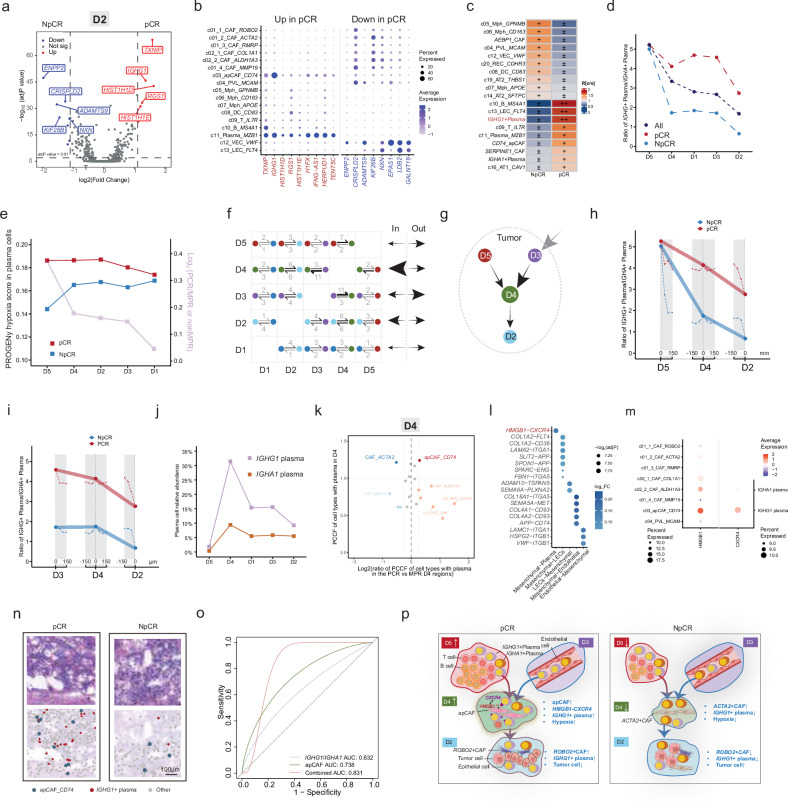
Multiomics Stamp-seq captures the treatment response-promoting spatiotemporal trajectory of plasma cells in NSCLC. **a** Volcano plot highlighting genes that are differentially expressed in nonepithelial cells within epithelial district D2 between pCR and NpCR samples. **b** Dot plot displaying representative differentially expressed genes (from **a**) across nonepithelial cells. The dot size represents the fraction of cells expressing specific genes, while the color intensity indicates relative expression levels. **c** Heatmap illustrating the sample preference of cell subsets within a 100 µm radius of neoplastic cells, quantified by the Ro/e score. **d** Line chart showing the ratio of *IGHG1*⁺ to *IGHA1*⁺ plasma cells across districts in total, pCR, and NpCR samples. **e** Line chart depicting hypoxia pathway activity, calculated using PROGENy, across districts in plasma cells in pCR and NpCR samples. **f** Clonal plasma cell transfer frequencies across districts, with summed values displayed on the right. “In” denotes movement into a district, and “out” indicates movement out of a district. The arrow thickness and accompanying numbers represent transfer frequencies (related to Supplementary Table S6). **g** Inferred plasma cell migration model based on panel f. The black arrows indicate the movement orientation, the gray arrow indicates the origin of D3 plasma cells from outside the tumor (gray dotted circle), and the arrow thickness reflects the transfer frequency between districts. Line charts displaying the ratio of *IGHG1*^+^ to *IGHA1*^+^ plasma cells in pCR and NpCR samples along the D5–D4–D2 (**h**) and D3–D4–D2 (**i**) routes. Dotted lines represent ratios at distances of 0, 0–50, 50–100, and 100–150 μm from district boundaries. **j** Line chart showing the relative abundance of *IGHG1*^+^ and *IGHA1*^+^ plasma cells across districts in pCR and NpCR samples. **k** Volcano plot of the spatial colocalization scores for various cell types with plasma cells in D4 of pCR and NpCR samples. **l** Dot plot of spatially co-occurring receptor–ligand interactions between sender–receiver pairs within a 100 μm radius, with a normalized *P* value < 0.005 (related to Supplementary Table S7). **m** Dot plot showing the expression of *HMGB1* in the CAF subclusters and that of *CXCR4* in the plasma cell subclusters. **n** Representative spatial map of Stamp-seq profiles of apCAFs and plasma cells onto the corresponding H&E-stained section of the profiled region. **o** ROC curve analysis of the GSE31245 dataset in which the relative GSVA scores of apCAFs, the *IGHG1*/*IGHA1* ratio, or their combination were used to predict responders. **p** Schematic model of the tumor fibroblastic microenvironment promoting spatiotemporal plasma cell movement across districts in NSCLC samples from patients who achieved a pCR or NpCR.

We analyzed the cellular origin of genes that were differentially expressed in non-epithelial cells within the D2 tumor region to determine the immediate tumor microenvironment dynamics related to the treatment response. Notably, genes that were upregulated in pCR samples were predominantly expressed by plasma cells, whereas NpCR samples were enriched in CAFs and endothelial cells (Fig. 5a, b). This spatial association suggested that plasma cells are immediate neighbors of tumor cells in favorable responders. Subgrouping plasma cells by *IGHC* expression revealed two distinct populations: 3431 *IGHG1*^+^ cells and 1304 *IGHA1*^+^ cells (Supplementary Fig. S10a). Interestingly, compared with their *IGHA1*^*+*^ counterparts, *IGHG1*^+^ plasma cells resided closer to tumor cells (Fig. 5c), consistent with prior reports that the infiltration of IgG^+^ plasma cells positively correlates with that of CD8^+^ T cells and improves immunotherapy outcomes in patients with NSCLC^26^, ovarian^28^, renal^29^, and bladder cancers^30,31^, whereas IgA^+^ plasma cells play immunosuppressive roles in prostate cancer^32^. Spatial profiling of the plasma cell distribution indicated higher *IGHG1/IGHA1* ratios in pCR samples across all districts, peaking in D5 (Fig. 5d and Supplementary Fig. S10b). We thus hypothesized that the mechanisms underlying *IGHG1*^+^ plasma cell generation and their tumor-targeted recruitment may drive antitumor immunity in patients who achieve a pCR.

We performed a comparative pathway analysis of pCR and NpCR samples to investigate the pathways that drive plasma cell generation and detected elevated hypoxia signaling in D5 regions of pCR tumors (Supplementary Fig. S10c). The expanded analysis revealed increased hypoxia activity across all lymphoid cells (T, B, and plasma cells) in the pCR samples district-wide (Fig. 5e and Supplementary Fig. S10d). Compared with *IGHA1*^*+*^ cells, *IGHG1*^+^ plasma cells exhibited stronger hypoxic responses (Supplementary Fig. S10e), with elevated glycolytic activity but no significant difference in oxidative phosphorylation activity (Supplementary Fig. S10f, g). Given the established role of hypoxia in plasma cell generation^33^ and that elevated glycolysis in the TME facilitates B-cell metabolism and promotes B-cell differentiation into IgG-producing plasma cells^34,35^, these findings imply that TLS-associated hypoxia may drive excessive plasma cell production, potentially increasing immunotherapy efficacy.

We developed a BCR spatial profiling technique to determine plasma cell clonotypes and their spatiotemporal trajectory (Methods; Supplementary Fig. S11a and Table S6). Utilizing probes targeting the CDR3 region, we captured and sequenced CDR3-containing cDNA molecules from the Stamp-seq cDNA library to assign clonotypes to plasma cells in the pCR sample. Next, we identified pairs of plasma cells that presented with the same clonotype but were located in different districts. In these plasma cell pairs, we determined the order of cellular movements between different districts using the cell state sequence inferred using the slingshot trajectory analysis and summed all the data (Supplementary Fig. S11b–d; Methods). BCR spatial profiling analysis revealed two migration routes: D5→D4→D2 and D3→D4→D2 (Fig. 5f, g and Supplementary Fig. S11). The χ2 test demonstrated that this migration pattern was not randomly distributed (*P* = 0.0452), indicating a directed and coordinated plasma cell migration process within the tissue architecture (Methods). Along the D5–D4–D2 axis, the pCR samples maintained high *IGHG1/IGHA1* ratios during transit, whereas NpCR samples showed an attenuation of this ratio starting in D4 and persisting in D2 (Fig. 5h). Conversely, the D3–D4–D2 route resulted in elevated baseline *IGHG1/IGHA1* ratios in D3 of pCR samples (Fig. 5i), indicating that external niches may pre-enrich *IGHG1*^*+*^ cells before tumor entry. Thus, D4 emerged as the primary site for the enrichment of *IGHG1*^+^ cells along both migration routes (Fig. 5j), suggesting its role as a “transit hub” prior to tumor infiltration in D2.

The treatment-response-related cellular neighborhood of plasma cells was calculated to investigate how D4 becomes enriched in *IGHG1*^*+*^ plasma cells. The cellular neighborhood analysis revealed that apCAFs were proximal neighbors to D4 plasma cells in pCR samples, in contrast to *ACTA2*^*+*^ CAFs in NpCR samples (Fig. 5k). Spatial interaction screening (within a 100 μm radius) revealed the *CXCR4* (*IGHG1*^+^ plasma cell)–*HMGB1* (apCAF) pairing (Fig. 5l and Supplementary Table S7). Considering that HMGB1 often acts as a co-factor to enhance CXCL12–CXCR4 signaling^36^, we further investigated the expression of *CXCL12* and found that *CXCL12* was expressed at significantly higher levels in cells proximal to *IGHG1*^+^ plasma cells than in those located farther away (*P* = 0.002, fold change = 1.15). Within fibroblast populations, *CXCL12* is a marker of inflammatory CAFs. We found that high *CXCL12*-expressing CAFs c02_2_CAF_*ALDH1A3* (Supplementary Fig. S12a) exhibited the highest spatial colocalization score with apCAFs within spatial domain D4 (Supplementary Fig. S12b). We further validated our findings using CellChat v2 to analyze cell–cell communication within 100 μm and discovered that the *HMGB1–CXCR4* signaling pathway is significantly activated between mesenchymal cells and plasma cells (Supplementary Table S10). Combined with the high expression of *HMGB1* in apCAFs across all mesenchymal cells and *CXCR4* in *IGHG1*^+^ plasma cells (Fig. 5m), we confirmed that *CXCR4*–*HMGB1*-mediated crosstalk occurred between apCAFs and *IGHG1*^+^ plasma cells. Colocalization of these subsets was pronounced in pCR tissues (Fig. 5n), mirroring known *CXCR4*-mediated plasma cell retention mechanisms in bone marrow niches^37^.

Public NSCLC datasets corroborated that apCAF signatures and high *IGHG1/IGHA1* ratios correlate with an improved therapeutic response (Supplementary Fig. S13a) and progression-free survival (PFS; Supplementary Fig. S13b, c). The combination of both the apCAF signature and the *IGHG1/IGHA1* ratio resulted in prolonged PFS, with an area under the curve (AUC) of 0.831 (Fig. 5o). Together, these results position D4 as a critical niche where apCAFs recruit *IGHG1*^+^ plasma cells, originating from TLSs (D5) or peripheral sources (D3), via HMGB1–CXCR4 interactions, facilitating their antitumor migration toward tumor cells (D2) in chemoimmunotherapy-responsive patients (Fig. 5p).

## Discussion

Here, we present the use of Stamp-seq, a spatial-snRNA profiling technique, to characterize treatment-response-related cell communities. Compared with the snRNA-seq data, the application of our technique to the adult mouse brain revealed precise transcriptomic capture, which assigned nuclear identity with high similarity and accurate spatial localization by mapping nuclei to different cortical layers and to microdomains such as endothelial cell-rich regions. We also applied Stamp-seq to samples from patients with NSCLC treated with neoadjuvant chemoimmunotherapy and identified treatment response-related spatial cell communities, including a TLS district and a plasma cell niche. Combined with spatial BCR profiling, we revealed the spatiotemporal recruitment of *CXCR4*^*+*^ *IGHG1*^*+*^ plasma cells from TLSs and blood circulation by *HMGB1*-expressing apCAFs in the plasma cell niche, ultimately leading to the enrichment of *IGHG1*^*+*^ plasma cells in the tumor cell region in good responders.

Stamp-seq represents a cost-effective platform for spatially resolved single-nucleus transcriptomics, with distinct advantages across critical parameters when benchmarked against existing technologies. In terms of resolution and performance, Stamp-seq achieves a 1.6 μm barcode density with < 3.2% duplication across an 85 mm² capture area, enabling nuclear localization within a half-cell diameter of true positions, surpassing multicellular-resolution methods such as DBiT-seq^3^ (10–50 μm) and 10× Visium (55 μm) while outperforming subcellular-resolution techniques such as Stereo-seq (0.5 μm), Seq-scope (0.5–0.8 μm) and Visium-HD (2 μm) that rely on computational boundary inference rather than direct mapping. In contrast to the nuclear-labeling approach, Slide-tags Stamp-seq uniquely provides on-chip gold-standard staining validation that is absent from the Slide-tags methodology. With > 99% spatial barcode recovery and ~79% location assignment efficiency, Stamp-seq achieves robust precision in nuclear mapping that is unattainable by conventional alternatives.

Regarding data quality, Stamp-seq detects 400–2500 genes per nucleus, exceeding most spatial methods, although it is still below Slide-tags, potentially due to its cross-linking step. Crucially, despite 60%–90% nuclear loss during extraction and microfluidics, captured nuclei exhibit transcriptional profiles that are more congruent with snRNA-seq references than Slide-tags, Stereo-seq, or DBiT-seq, confirming biological fidelity.

Regarding sample compatibility and turnaround time, nuclear extraction from frozen sections in Stamp-seq bypasses the perfusion requirements of Visium and Stereo-seq, supporting any tissue amenable to nuclear isolation. Library preparation requires approximately one day plus standard NGS sequencing time, which is faster than imaging-based workflows and is competitive with other sequencing-driven platforms. Its sequencing-by-synthesis fabrication further accelerates processing compared with bead-based (Slide-seq, and Slide-tags) or DNA nanoball-based (Stereo-seq) array generation, establishing a practical throughput advantage for large-scale studies.

With respect to economic efficiency, Stamp-seq achieves *a* > 10× reduction in library preparation costs by processing 100,000+ cells per reaction compared with conventional methods (10,000–20,000 cells), while replacing expensive prefabricated microarrays with sequencing-by-synthesis-fabricated spatial chips, slashing per-chip costs to near-standard NGS flow cell levels.

Regarding modality extensibility, Stamp-seq demonstrates seamless compatibility with diverse nuclear profiling methods, including B-cell clonotype mapping (as exemplified here), and supports applications ranging from sn-ATAC-seq to sn-methylation profiling, among others. This flexible integration of multiomics workflows constitutes a unique advantage of nuclear-labeling approaches represented by Stamp-seq and Slide-tags.

The main caveat of Stamp-seq is the general loss of direct-contact cell partners due to unavoidable nuclear loss. The overall nuclear recovery rate, calculated as the ratio of localized and sequenced nuclei to DAPI-stained nuclei outlined on-chip, ranged from approximately 10% in necrotic or fibrotic tumor samples to 37% in adult mouse brain sections (see “Methods”). This variation in efficiency stems from cumulative losses at two critical stages: nuclear extraction techniques routinely incur 15%–40% nuclear loss because of mechanical and enzymatic dissociation constraints, whereas microfluidic handling contributes to an additional 33%–68% loss (Supplementary Table S9). Enhancing nuclear extraction techniques and minimizing microfluidic loss can reduce loss to a certain degree. Moreover, combining in situ cytoplasmic RNA capture and nuclear labeling on the same chip could serve as a method development direction. Another caveat is the generally lower gene detection depth compared with that of snRNA-seq. This limitation stems primarily from the pre-extraction fixation protocol; while this step increases spatial barcode retention efficiency and facilitates extended on-chip nuclear imaging, it concurrently induces RNA fragmentation, thereby compromising transcriptome integrity and reducing the number of detectable genes. Further improvements could be achieved by performing reversible fixation with reagents such as DSP^38^. Additionally, we are developing an improved protocol incorporating a probe-based hybridization strategy, similar to snPATHO-seq^39^, where oligonucleotide probes directly hybridize to target RNA sequences independent of reverse transcription, to overcome this technical limitation, thereby increasing detection sensitivity in crosslinked single-cell samples.

Spatially resolved information at true single-cell resolution could be instrumental for segregating cellular communities. Immunotherapy represents a paradigm shift for the treatment of malignancies across a broad range of indications, but not all patients benefit from such therapy. A powerful predictive biomarker can optimize treatment choices and identify likely beneficiaries. In addition to single-module biomarkers such as PD-1/PD-L1 expression, spatial cell communities can be prognostic markers. For instance, the presence of TLSs is a better prognostic biomarker in nearly all cancers^40^. An increased density of plasma cells is associated with more favorable outcomes in several cancers, including NSCLC^26^ and soft-tissue sarcomas^41^, at least partly through the secretion of IgG that binds to tumor cells^29^ and is associated with CD8^+^ T-cell infiltration^28^. Although the majority of plasma cells are observed to intratumorally localize to regions that are not TLSs^28^, the cellular composition of such regions remains unknown. Previous studies have shown that plasma cells lodging in the bone marrow are long-lived and secrete IgG and that the bone marrow survival niche contains stromal cells that express *CXCL12*, which anchors *CXCR4*^*+*^ plasma cells to promote survival^42,43^. Our findings suggest that a comparable specialized niche for the survival of IgG-secreting plasma cells may exist locally within tumors, and this niche likely has a connection to a preferred immunotherapy response. In this niche, apCAFs are key mesenchymal cells that specifically recruit *CXCR4*^*+*^ *IGHG1*^*+*^ plasma cells by expressing HMGB1, a known cofactor of CXCL12, to promote its binding to CXCR4^36^. Consequently, based on our cell community analysis, the apCAF signature alone or together with the *IGHG1/IGHA1* ratio is a good prognostic marker for the efficacy of chemoimmunotherapy against NSCLC.

In addition to forming a niche for the survival of plasma cells, fibroblasts also closely contact other cells. We found that *ROBO2*^*+*^ CAFs localized to the periphery of tumor cells and TLSs in samples from patients who achieved a pCR, and that *ACTA2*^*+*^ and *COL1A1*^*+*^ myCAFs were enriched in the periphery of tumor cells in samples from patients who achieved an NpCR. A recent study indicated that *COL11A1*^*+*^ CAFs can deposit collagen, obstruct T-cell infiltration, and lead to a poor prognosis for NSCLC^44^. Our data revealed that *COL11A1* is highly expressed in both *ACTA2*^*+*^ and *COL1A1*^*+*^ myCAFs (Supplementary Fig. S9b), suggesting that the spatial proximity between myCAFs and tumor cells in NpCR samples could lead to the exclusion of T cells and a lack of a chemoimmunotherapy response. Together, our findings indicate that spatial communities containing fibroblasts have a pleiotropic effect on multiple elements of the TME to modulate the treatment response. Our findings also indicate that methods designed to delineate spatially resolved cellular states promote the development of diagnostic and prognostic modules for human diseases.

## Methods

### Mouse and human sample information and processing

#### Mouse brain

Wild-type C57BL6/J mice, aged 6 to 8 weeks, were procured from Beijing Vital River Laboratory. Their brains were carefully dissected and immediately embedded in Tissue-Tek OCT (Sakura, 4583), followed by transfer to a –80 °C freezer for long-term storage. All procedures involving animal experiments described in this study adhere to ethical regulations governing animal research.

#### Human non-small cell lung cancer samples

Specimens were acquired from patients who underwent lung tumor resection surgeries for non-small cell lung carcinoma after combined neoadjuvant chemotherapy and anti-PD-1 antibody (JS001) treatment at Hunan Provincial Tumor Hospital in China as part of a stage II investigator-initiated clinical trial, which was approved by the ethics committee under identification number JS001-ISS-CO147. The treatment response was evaluated by two pathologists based on tumor section histology. Tumors were categorized as pCR, MPR and non-MPR. pCR represents a failure to detect viable tumor cells, MPR represents less than or equal to 10% viable tumor cells, and non-MPR represents greater than 10% viable tumor cells. The specimen was snap frozen following surgery and stored at –80 °C until embedding in tissue-Tek OCT (Sakura, 4583) and sectioning using cryostats. The use of tissue at the Beijing Institute of Genomics at the Chinese Academy of Sciences was approved by the Institutional Review Board under project number 2023H039.

#### Coculture of human and mouse cells

The K562 cell line (ATCC, CCL-243) and the YAC-1 cell line (ATCC, TIB-160) were obtained from ATCC and cultured in accordance with ATCC guidelines. Freshly harvested cells were washed with 1× PBS and resuspended at a concentration of 1 × 10^6^ cells/ml for the species mixture experiment.

### Overview of Stamp-seq technology

1 Stamp-seq chip (SeekSpace® chip) synthesis and manufacture

#### Generation of the Seed Oligo Library (Supplementary Fig. S1a, S1b.1)

Stamp-seq was initiated with the generation of a single-stranded DNA oligo seed library with 32 random nucleotides that served as spatial barcodes.

Library structure:

5′ P5 (Uracil containing)-Rd2P binding site-Spatial barcode-Spatial barcode seq primer binding site-P7 3′

Stamp-seq ssDNA sequence:

5′AATGATACGGCGACCACCGAGATCTACACGTGACTGGAGTTCAGACGTGTGCTCTTCCGATCTNNVNBVNNVNNVNNVNNVNNVNNVNNVNNNNNTCTTGTGACGACAGCACCAAAAAAAAAAAAAAAAAAAAAAAAAAAAAATTTGACTTTCACCAGTCCATGATATCTCGTATGCCGTCTTCTGCTTG3′

#### Oligo cluster generation and sequencing (Fig. 1a*,* steps 1 and 2*;* Supplementary Figs. S1a, S1b.2, 3, 4)

Using GenoLab M protocols, Stamp-seq oligo clusters were generated by 28 cycles of amplification of the oligo seed library at a concentration of 2.8 pM. The oligo clusters were subsequently sequenced on the GenoLab M sequencing platform using Seqprimer-4 as the custom primer. The Seqprimer-4 sequence is provided below. Sequencing reactions were performed for 34 rounds. In the first 32 rounds, the spatial barcode region was sequenced, and rounds 33 and 34 were used for quality control and cluster density evaluation. Images of the clusters were generated using the GenoLab Control System (GLCS; Supplementary Fig. S1b). The oligo clusters were then washed three times with water, three times with 0.1 N NaOH, three times with 0.1 M Tris (pH 7.5), and three times with water.

The spatial barcode region contains 32 random nucleotides, which can produce 1 quintillion different sequences. We observed a duplication rate of less than 0.1% at a concentration of 2.8 pM (Supplementary Fig. S1d). The duplicated reads were confidently removed in the data analysis step (see below).

Seqprimer-4:

5′ATCATGGACTGGTGAAAGTCAAATTTTTTTTTTTTTTTTTTTTTTTTTTTTTTGGTGCTGTAGTCACAAGA3′

#### Processing flow cells into the Stamp-seq array (Fig. 1b)

A laser segmentation machine (Inte Laser, MLC-10ZW) was used to cleave the flow cell coated with oligo clusters into 5.5 mm × 15.5 mm regions. We adhered two capture regions onto a glass slide to complete the Stamp-seq chip (SeekSpace® chip) assembly. Each Stamp-seq chip library contains information for both the spatial barcode sequencing results and the *x* and *y* locations for each spatial barcode cluster.

2. Stamp-seq sample processing

Cryosections were cut at a thickness of 10 μm or 20 μm using a Leica CM1950 cryostat to be mounted onto the Stamp-seq chip for further processing. Mouse brains were sectioned coronally, and human lung tumors were sectioned to their largest area. Neighboring 10 μm sections were also mounted onto new glass slides, stained with H&E, and imaged under brightfield using a Leica DMi8 microscope.

3. Cell nucleus tagged with spatial barcode preparation

Single-cell nuclear suspensions with spatial barcodes were prepared from fresh frozen tissues using the SeekSpace® Single-Cell Spatial Transcriptome-seq Kit (K02501-08; Fig. 1a, steps 3 and 4; Fig. 1c, step i). Briefly, tissue regions of interest were placed on the Stamp-seq chip, ensuring that no folds were present. A finger was placed on the back of the Stamp-seq chip to melt the tissue. The Stamp-seq chip was then placed in a chip holder and incubated on a thermocycler adapter at 37 °C for 90 s. After 90 s, a chamber was placed on the chip, and 150 µL of labeling reagent (10 mM Tris-HCl (pH 7.4), 10 mM NaCl, 3 mM MgCl_2_, 1 U/µL RNase inhibitor, 0.1% IGEPAL CA-630, and 20 U/mL USER® enzymes) was added without introducing bubbles. Next, the tissue sections were fixed with 150 µL of 4% formaldehyde containing 1 µL of ssDNA dye for 12 min and simultaneously imaged using a Leica DMi8 fluorescence microscope. The samples were subsequently homogenized in prechilled lysis buffer (10 mM Tris-HCl (pH 7.4), 10 mM NaCl, 3 mM MgCl2, 1 U/µL RNase inhibitor, and 0.1% IGEPAL CA-630) using a Dounce homogenizer (KIMBLE #D8938). After the cells were washed with PBS containing 1 U/µL RNase inhibitor and filtered through a 70 µL cell strainer, the number of nuclei was estimated using a fluorescence cell analyzer (SeekGene Tinitan® Fluorescence Cell Counter or Countstar® Rigel S2) with AO/PI before being placed on ice for further analysis. Spatial chip nuclear dissociation yields 100,000–200,000 nuclei across diverse human and murine tissue types, a throughput range ideally suited for multiplex barcoding workflows (Supplementary Table S9).

4. Sequencing library preparation

#### snRNA-seq library preparation

A single-cell RNA-seq library and spatial barcode library were prepared using the SeekSpace® Single-Cell Spatial Transcriptome-seq Kit (K02501-08) according to the manufacturer’s instructions. Briefly, the nuclei were evenly divided into 8–16 PCR tubes, and reverse transcription was performed on 600–30,000 nuclei in each PCR tube, with a differentially labeled reverse transcription primer (termed multiplex barcode; Supplementary Table S8) added to each tube (Fig. 1c step ii). Fifteen cycles of annealing (ramping from 8 °C to 42 °C) were performed to increase primer hybridization and intracellular reverse transcription efficiency. After reverse transcription, the nuclei were washed twice to remove residual primers and pooled together. Subsequently, up to 320 thousand nuclei were combined with T4 ligase in 1*×* ligation buffer (NEB M0202L) and bridge-oligo (AGCAACGACGGACGACAGCAA) and then added to the sample wells of the SeekOne® DD Chip S3 (Chip S3). Barcoded hydrogel beads (for detailed information, refer to BHBs in scFAST-seq^45^ with the sequence CTACACGACGCTCTTCCGATCT(j)17(N)12TTGCTGT, where (j)17 represents the 17 bp cell barcode sequences and (N)12 represents the random 12 bp sequences) and partitioning oil were then dispensed into the corresponding wells separately in Chip S3. The cell-containing ligation reagents and BHBs were encapsulated into emulsion droplets using the SeekOne® Digital Droplet System. Immediately after the emulsion droplets were transferred into PCR tubes, a 60-min incubation at 20 °C followed by a 10-min heat inactivation at 65 °C was performed to obtain barcoded cDNA and spatial barcodes (Fig. 1c, step iii). The barcoded cDNA and spatial barcodes were then decrosslinked with 2*×* lysis buffer (20 mM Tris (pH 8.0), 400 mM NaCl, 100 mM EDTA (pH 8.0), and 4.4% SDS) and 10 μL of proteinase K solution (20 mg/mL) and recovered from the cells in the droplets (Fig. 1c, step iv). A second round of reverse transcription was performed, followed by PCR pre-amplification to obtain more template-switched cDNA (Fig. 1c, step v). The pre-amplified product was used as input for both spatial barcode library construction and cDNA construction. Finally, sample indices were added to the pre-amplified product during spatial barcode library construction via PCR (Fig. 1c, step vi). After cDNA purification, 20 ng of cDNA was amplified by index PCR. The indexed sequencing libraries were subsequently purified using AMPure beads and quantified by quantitative PCR (KAPA Biosystems KK4824).

#### CDR3 enrichment and sequencing

For the enrichment of single-cell CDR3 regions, the cDNA libraries from sample T24, which were amplified from the pre-amplification product, were hybridized with 5’ biotin-labeled probes targeting the C regions of BCR and TCR genes (see the hybridization capture probes in Engblum et al.^46^), captured using M270 streptavidin beads and amplified using TSO and Read1 sequencing primers in accordance with the manuscript (Customized from Boke Bioscience). The target products were fragmented, end-repaired, “A” was added, and ligated to the Illumina TruSeq adapter using T4 DNA fast ligase. The ligation products were purified using 0.6 *×* DNA clean beads and amplified in a 50 μL assay with 25 μL of 2*×* KAPA HiFi HotStart Ready mix and 2 μL of 10 μM index primers. After an incubation at 98 °C for 3 min, 8 PCR cycles were performed (98 °C for 20 s, 54 °C for 30 s, and 72 °C for 20 s). The final CDR3 library was purified by 0.9*×* DNA clean beads.

#### Sequencing

The single-cell RNA-seq library, spatial barcode library and CDR3 library were subsequently sequenced on the Illumina NovaSeq 6000 platform with a PE150 read length or the DNBSEQ-T7 platform with a PE150 read length.

### Stamp-seq data preprocessing

#### snRNA-seq data

We utilized SeekSpace® Tools bcf2fastq to generate demultiplexed FASTQ files from the raw sequencing reads and aligned these FASTQ files to either the human GRCh38 or mouse mm10 genome to create a filtered feature-barcode matrix for each sample. Barcodes include multiplex barcodes and droplet barcodes. The “forceCell” method was employed with the default settings during the cell calling process. UMI counts for the top 80,000 cells were extracted, with a default threshold set at a minimum of 200 UMIs. Only those cells with UMI counts exceeding this threshold were selected as the final determined cells, which were subsequently used to generate the expression matrix.

#### Spatial barcode library preprocessing

Some of the spatial barcodes extracted from the spatial barcode library may be invalid. These invalid barcodes may arise from the inclusion of shorter mRNA fragments from the RNA library. In addition to this factor, sequencing errors can also result in invalid barcodes. We filtered out the invalid spatial barcodes that were absent from the Stamp-seq chip library to ensure the accuracy of the data.

Furthermore, a small percentage of spatial barcodes may have appeared multiple times with different coordinates, resulting from rare oligo synthesis duplication events. Since we could not determine the exact spatial location of these barcodes, they were also filtered out.

Additionally, certain spatial barcodes exhibited anomalously high UMI support. We hypothesized that they may have detached from the chip during experimental procedures and been encapsulated by many droplets, rendering these spatial barcodes inaccurate and prevalent. Moreover, cells carrying these exceptionally high spatial barcode UMIs could be mislocalized and should be removed from further analysis. We implemented the following steps to address this issue:

(i) The 5.5 *×* 15.5 mm chip was divided into bins of size 30 pixels *×* 30 pixels. (ii) The total number of UMI supports was counted for all spatial barcodes in each bin. (iii) Bins were sorted in descending order of UMI support. (iv) The threshold (20 times the average UMI count of all spatial barcodes within each bin) was calculated based on the distribution of the sorted bins. (v) If the UMI support of a bin exceeded the threshold, we removed the cell with the highest spatial barcode UMI support in that bin, as well as all other spatial barcodes associated with that cell. This approach resulted in the clearance of the most affected cell and freed other cells from the influence of the mislocalized prevalent spatial barcode in this bin. Finally, we filtered out all cell barcodes not associated with biologically meaningful cells and their corresponding spatial barcodes, focusing solely on locating cells of biological significance.

#### Retrieval of spatial barcodes and associated coordinates

The first step in acquiring the spatial *x*, *y* coordinates for Stamp-seq nuclei is to retrieve all spatial barcodes related to each nucleus and their coordinates. This analysis required two distinct libraries, namely, the spatial barcode library and the Stamp-seq chip library. The UMIs of the spatial barcode library represent the expression level of each spatial barcode. The Stamp-seq chip library contains single-end sequences with a read length of 32 bases linked to specific spatial coordinates (Fig. 1b; Supplementary Fig. S1a). Corresponding spatial barcodes were extracted from the Stamp-seq chip library to establish the correspondence between cell barcodes (defined as a combination of spatial barcodes and cell barcodes) and spatial barcodes.

When a spatial barcode extracted from the spatial barcode library matched a barcode extracted from the Stamp-seq chip library (i.e., the whitelist), it was considered valid, and its associated reads were counted. Otherwise, it was classified as invalid. Notably, sequencing errors may occasionally occur. SeekSoul® Tools supports barcode correction by comparing invalid barcodes to the spatial barcode whitelist. An invalid barcode with a single-base mismatch (hamming distance of 1) was corrected to the corresponding barcode in the whitelist when a single match was identified. In cases where multiple matches were found, the correction was made to the barcode with the highest read counts. A similar correction was performed for the multiplex barcodes and droplet barcodes. Cells with corrected barcodes were then retrieved again, along with their coordinates.

#### Determination of the cell position

The centroid position of the cell was equal to the centroid position of the nucleus in this study. When determining the centroid position of a nucleus, the presence of noisy spatial barcodes must be considered. These barcodes may persist as background signals within droplets during experiments or be marked on nuclear fragments, leading to multiple centroid positions on the chip associated with the same cell barcode. Consequently, nuclei with multiple centers must be filtered out to ensure that only those with clearly defined centroids are retained.

As shown in Fig. 2d, the spatial barcode distribution of a nucleus (for which at least 2 spatial barcodes UMIs are detected) includes three scenarios: a unique center and multiple centers with and without a major center.

In the panels on the right side of Fig. 2d, each grid represents a bin, measuring approximately 100 pixels (26.5 μm) per side, with an area of approximately 0.02 mm^2^. The color intensity of each bin indicates the number of UMIs for all the spatial barcodes located within that bin, with deeper hues signifying higher UMI counts. The darkest blue bin indicates the bin with the highest UMI support for this cell, which is identified as the core center. This center bin, along with the 24 immediately adjacent bins, is collectively referred to as the core of the cell.

We introduced the concept of a secondary center, which is defined as the bin exhibiting the highest UMI support outside of the core, to determine whether a cell possesses multiple centers. We calculated the ratio of the total UMI count within the core to that within the secondary center and its surrounding 24 bins. A ratio equal to or exceeding 10 characterized the cell as having a unique center; a ratio equal to or exceeding 2 characterized the cell as having a major center; and a ratio smaller than 2 classified the cell as having multiple centers.

Afterward, we identified the spatial coordinates of cells with either a unique center or a major center. We calculated the spatial coordinates of each cell by determining the nearest spatial point to all identified spatial barcodes within the unique cell core or the major cell core (25 bins in total, including the 24 surrounding bins and 1 center bin), measured using Euclidean distances.

Moreover, we computed the distribution of Euclidean distances between each detected spatial barcode and the final spatial coordinates established for each cell in the core region. We then assessed the distribution of distances for the closest 50%, 75%, and 95% of spatial barcodes to evaluate the dispersion of spatial barcodes (Supplementary Fig. S2c).

### Mouse brain analysis

#### Quality control and cell type assignment

For sample WTH1092, the filtered feature–barcode matrix was generated using SeekSpace® Tools and subsequently imported into R (v.4.1.1) using Seurat (v.4.0.6). For sample WTH1092, after the quality control step, we identified a total of 42,499 cells with more than 300 UMIs and less than 5% mitochondrial gene content, 42,317 of which were combined with spatial barcodes, using SeekSpace® Tools. After removing cells classified as “scratched/contaminated” from those with spatial barcodes, we retained 41,947 cells. A total of 41,641 of these cells, each with a spatial barcode count of ≥ 2, were analyzed to determine the presence of a unique spatial center. During this analysis, 17,259 cells exhibited the highest spatial barcode centrality-to-secondary centrality ratio of ≥ 10, while 15,778 cells had a ratio between ≥ 2 and < 10, and 8604 cells had a ratio of < 2. Ultimately, we identified 33,037 cells as having a discernible unique center based on their spatial barcodes, with 32,833 of these overlapping with the tissue area on the DAPI image. This process resulted in a matrix comprising 32,833 nuclei, with a median of 738 UMIs per cell and 421 median genes per cell. Cell cycle scoring was performed using the “CellCycleScoring” function. We utilized the “SCTransform”^47^ function for normalization and variance stabilization, which concurrently normalizes the data and identifies highly variable genes. This method involves the regression of confounding sources of variation, including mitochondrial percentage and cell cycle scores. We selected 2000 variable features for subsequent analyses. Following normalization, dimensional reduction was executed using principal component analysis on the top 2000 variable genes. The first 30 principal components were employed for UMAP to visualize the cells in two-dimensional space. Graph-based clustering was subsequently conducted at a resolution of 0.4 to identify distinct cell populations, with each cell cluster being validated using known marker genes. Note that the cDNA library includes sequences from mRNAs (~59.3%), lncRNAs (~3.1%), rRNAs (~28.4%), mtRNAs (~6.96%) and others (~2.31%) in WTH1092 because of the randomized primers used in the RT reaction in step ii.

#### Assessment of the nucleus capture efficiency using multiplex barcodes

Considering that a substantial number of cells were filtered out during spatial cell localization because of the inability to assign a unique or major spatial center when multiple centers were detected, we proposed a novel principle aimed at “recovering” these discarded cells and increasing cell capture. In this method, nuclei were uniformly divided into multiple subgroups prior to the formation of water-in-oil emulsions, with distinct multiplex barcodes applied to each subgroup (Fig. 1a).

Without the application of multiplex barcodes, a cell that exhibited multiple centers, which could be due to multiple cells being together or a cell and debris coexisting in the same droplet, would be discarded. The addition of multiplex barcodes reduced signal interference among cells and debris.

We determined the optimal number of multiplex barcodes required to maximize the recovery of discarded cells due to multiple centers by conducting simulations on the WTH1092 sample using 1, 2, 4, and 8 multiplex barcodes. When only one multiplex barcode (equivalent to not applying any multiplex barcode) was employed, 27,561 cells were defined. With two multiplex barcodes, the number of cells with a definitive spatial center increased to 30,177. With four multiplex barcodes, this number increased further to 31,808, and with eight multiplex barcodes, the number of cells mapped to a clear spatial center reached 32,828 (Supplementary Fig. S2a). Notably, this pattern suggests that the use of multiplex barcodes effectively increases the recovery of discarded cells. However, upon the use of eight multiplex barcodes, the cell rescue rate began to plateau, indicating a saturation point in recovery efficiency; hence, eight multiplex barcodes were selected for the Stamp-seq pipeline.

#### Determining the density and cluster width of spatial barcodes

We calculated the average interstitial distance between the centroids of spatial fluorescence signal clusters based on the number of clusters per unit area (a median value of 1.64 μm) to quantify the spatial barcode density. We subsequently used ImageJ (v1.52k) to measure the area of each cluster on the fluorescence image from cycle 33 of spatial barcode sequencing. Assuming that each spatial signal cluster was circular, we inferred the diameter of the clusters from their average area (a median diameter of 1.35 μm) on 100 equal segments, with each having an area of 0.1 mm^2^ (Supplementary Fig. S1c).

#### Qualification of the RNA composition

Sequencing reads were aligned to a mm 10 reference annotation file using STAR (v2.7.10a) with the default parameters and stringent mapping filters (--outFilterScoreMinOverLread 0.66). Gene-level quantification was performed using featureCounts (v2.0.3) with the default parameters, thereby enabling the proportional classification of RNA types (e.g., rRNAs, mRNAs, and lncRNAs) based on the annotated GTF features.

#### Comparison of the distance between Stamp-seq spatial localization assignment and actual nuclear coordinates on the DAPI image

We employed CellProfiler (v4.2.6) software to analyze the spatial coordinates of DAPI-stained nuclei within a specified region in the cortex (252 nuclei based on DAPI staining; 92 nuclei detected by Stamp-seq) of the mouse brain and determine the precision of Stamp-seq in determining the spatial localization of nuclei, resulting in a 37% nuclear capture rate. We performed coordinate axis transformation to compare the localization of nuclei from Stamp-seq spatial localization assignment and DAPI images by leveraging 14 manually selected pairs of spatial coordinate points as true references, and then utilized the MASS (v7.3.58.3) package in R to transform the coordinates. This calibration was necessary to ensure congruence between the axes of Stamp-seq and those rendered by CellProfiler. We calculated the number of nearest neighboring nuclei within the DAPI dataset for each nucleus identified by Stamp-seq. By defining these proximities as one cell-to-one cell correspondences, we ensured a unique DAPI nucleus pairing for each nucleus observed in Stamp-seq.

We subsequently simulated a scenario of randomness in spatial coordinates by generating spatial coordinates for 252 nuclei randomly. Based on the aforementioned methodology, we computed their nearest DAPI-stained nuclei. This method allowed us to compare the spatial localization accuracy of Stamp-seq with that of the randomly generated coordinates, gauging their respective distances to the actual DAPI-stained nuclei.

#### Identification of layers and layer-dependent gene expression

The laminar assignment of each excitatory neuron (encompassing layers L2–3, L4, L5, L5-6, and L6) was achieved using a two-step procedure. Initially, subpopulations of excitatory neurons were discerned through the expression of the pan-excitatory neuronal marker *Tbr1*. The laminar identity of each neuron was subsequently defined by the use of layer-specific markers (Supplementary Fig. S3d). Numerical laminar coordinates were calculated for each cell to quantify the spatial arrangement of excitatory neurons within each cortical layer. These coordinates were derived from the Euclidean distance between each cell and its nearest neighbor cell at the layer boundary.

#### Comparison of Stamp-seq snRNA-seq data and conventional snRNA-seq data

For the WTH1059 sample (Supplementary Fig. S4a), the SeekSpace® Tools were executed as previously described. This process yielded a comprehensive matrix consisting of 17,900 nuclei, with a median count of 913 UMIs and 539 genes per cell. The filtered feature–barcode matrix produced using SeekSpace® Tools was then imported into R (v.4.1.1) using the Seurat package (v.4.0.6). We investigated the correspondence between cell type distributions in Stamp-seq snRNA-seq data and conventional snRNA-seq data by integrating a reference dataset of the adult mouse brain^48^ alongside Stamp-seq snRNA-seq data using the “IntegrateData” function. Normalization and variance stabilization were conducted using the “SCTransform”^47^ function with the default parameters, enabling the concurrent normalization and identification of highly variable genes. Following the correction for variance-stabilizing transformation, we identified the top 2000 highly variable genes. Gene expression levels were subsequently scaled and centered. For two-dimensional visualization, nuclei were subjected to UMAP using the top 30 principal components, which were also employed to determine shared nearest neighbors. Through a comparative analysis of cell type proportions within the integrated snRNA-seq subpopulations, we identified eight distinct cell types: oligodendrocytes (Oligo), inhibitory neurons (Inh), astrocytes (Astro), excitatory neurons (Ext), oligodendrocyte precursor cells (OPCs), endothelial cells (Endo), microglia (Micro), and neuroblasts (Nbs). Finally, we utilized Spearman’s correlation analysis to assess the similarities between Stamp-seq snRNA-seq data and public snRNA-seq in terms of the cellular composition, mean number of features (nFeatures), and mean UMIs within each cell type (Supplementary Fig. S4b).

#### Comparison of Stamp-seq and snRNA-seq with Slide-tags, Stereo-seq, DBiT-seq, and snRNA

The performance of Stamp-seq in adult mouse brains was benchmarked against publicly available datasets produced using Slide-tags^9^, Stereo-seq^4^, DBiT-Seq^3^ (GEO: GSM4096261 in GSE137986), and snRNA-seq^19^. Stamp-seq, Slide-tags snRNA-seq, and snRNA data were processed, and nuclei were embedded in the UMAP space, as described above. Total UMIs from Stereo-seq (33 × 33, 10 μm) and DBiT-seq (10 μm resolution) spatial spots were normalized to 10,000 and log-transformed to report gene expression. The top 2000 highly variable genes were identified using variance-stabilizing transformation correction^49^. Gene expression levels were then scaled and centered. We identified shared nearest neighbors using the top 30 principal components. For two-dimensional visualization, we embedded spatial spots in the UMAP space utilizing the top 30 principal components, with a neighbor count of 30. Dot plots were constructed to display the normalized expression levels of the cell markers in each cluster.

We investigated the extent of potential contamination among transcripts from different types of cells by comprehensively analyzing the overlap of gene expression using cell type-specific markers, including *Rofox3*, *Csf1r*, *Slc1a3*, *Plp1*, *Flt1*, *Cspg4*, and *Ttr*. We evaluated the degree of overlap between any two markers by calculating the overlap ratio, defined as the ratio of the number of cells coexpressing both genes to the greater number of cells expressing either of the two genes individually. Mathematically, it can be formulated as follows:

$${Overlapratio}=\frac{{NumberofcellsexpressingbothgeneAandgeneB}}{\max ({NumberofcellsexpressinggeneA},{cellsexpressinggeneB})}$$

Additionally, we reasoned that some of these cell type markers are coexpressed in the same cell; thus, we calculated the similarity of the marker overlap ratio between snRNA-seq (representing the gold standard) and each platform (Stamp-seq, Slide-tags, Stereo-seq, and DBiT-seq) using the Pearson correlation coefficient (Supplementary Fig. S4r).

### Non-small cell lung cancer analysis

#### Quality control and cell type assignment

SeekSpace® Tools was employed to produce the filtered feature–barcode matrix for each slide. Matrices from all patients were subsequently analyzed utilizing the Seurat package^17^ (v4.0.6) within the R programming environment (v4.1.1). Cells with fewer than 300 UMIs or with more than 5% of the reads mapping to mitochondrial genes were excluded from further analysis. For normalization, the total UMIs per nucleus were scaled to 10,000, followed by log-transformation to report gene expression levels. We identified the top 2000 highly variable genes after applying variance-stabilizing transformation correction. Cell cycle scores, comprising those for the G1, G2/M, and S phases, were calculated using the “CellCycleScoring” function. All gene expression levels were scaled and centered, with the parameters set to regress “percent.mt”, “S.Score”, and “G2M.Score”. Batch correction was conducted within each cell compartment using the RunHarmony() function from the Harmony package^50^, with the key batch parameter “group.by.vars” assigned to “Sample”. For two-dimensional visualization, nuclei were embedded in a UMAP using the top 30 principal components. DoubletFinder^51^ (v2.0.3) was employed to detect doublet cells based on the matrices filtered by Seurat. Clustering of similar cells was performed using the FindClusters() function with a resolution set at 0.8. Differentially expressed genes (DEGs) across various clusters were identified using the FindAllMarkers() function. Annotation of the de novo clusters was aided by traditional marker genes.

#### Spatial receptor–ligand prediction

We employed a receptor–ligand score calculated using a spatial correlation index (SCI) to identify receptor–ligand interactions between pairs of cell types^52^. The expression data were processed with SCTransform counts^47^. We constructed a spatial weight matrix with dimensions N × M, where N denotes the number of sender cells, and M represents the number of receiver cells. This adjacency matrix is assigned a value of 1 if sender cell i is within 100 μm of receiver cell j and 0 otherwise. Initially, we utilized LIANA^53^ (v0.1.12) to generate a hypothetical list of receptor–ligand interactions between cell types, independent of the spatial context. This list was refined to include interactions present in at least 10 sender cells or in 10% of both sender and receiver cells. We subsequently calculated a spatial correlation index for each interaction to evaluate the spatial coexpression of receptors and ligands within these cell type pairs.

An adaptive spatial permutation test, involving 1000 permutations per interaction, was employed to assess the spatial significance of each receptor–ligand score by randomizing cell positions within the same cell type. We implemented the Benjamini–Hochberg correction to address multiple hypothesis testing. Additionally, we computed the log-transformed fold change (FC) between the observed SCI statistic and the median SCI statistic of the empirical null distribution, allowing comparisons of log-transformed FC values for the SCI across various receptor–ligand interactions without explicit correction for cell type frequencies. Ultimately, spatial cell communication pairs exhibiting a normalized *P* value of less than 0.005 were identified as genuine spatial interactions between the two cell types.

#### Inferring the CNV

Copy number variant profiles were estimated for each epithelial cell using the inferCNV R package (https://github.com/broadinstitute/inferCNV). Nonepithelial cell types, specifically, lymphatic endothelial cells (LECs) and vascular endothelial cells (VECs), were selected as references. The “infercnv::run” function from infercnv (v1.16.0) was employed with the following parameters: cutoff = 0.1, cluster_by_groups = FALSE, HMM = TRUE, analysis_mode = subclusters, denoise=T, HMM_type=i6, and tumor_subcluster_partition_method = random_trees.

#### Quantitative assessment of the cellular subtype distribution across tissues

We utilized the epitools R package (v0.5.10.1) to compare the observed versus expected cell counts for all cell types and subtypes and to investigate the potential enrichment of cellular subtypes across different regions or sample types. The ratio of observation to expectation (RO/E) was calculated as follows: Ro/e = observed/expected, where the expected number of cells was derived using the chi-square test. A specific cluster was deemed enriched within a particular tissue if Ro/e > 1.

#### PCCF statistic

We conducted a quantitative assessment of cell contacts and colocalization among various cell types using pairwise cross-correlation function (PCCF) statistics, as previously described for analyzing cellular colocalization^54^. In this analysis, the PCCF is defined as the ratio of observed colocalization events to the expected occurrences of random colocalization. We estimated the average number of random colocalization events by randomizing the original positions of cells and counting the occurrences of touching and overlapping between cells over 100 simulations. Two cells were considered genuinely colocalized if they were within a spatial distance of 30 μm. By tallying the number of colocalization events between different cell types, we calculated the PCC value for each pair of cell types using the following formula:

$${PCCF}=\frac{{Observed}({colocalizatedpairnumber})}{{Expected}({meancolocalizatedpairnumber})}$$

#### Unsupervised cellular districting using GraphSAGE

We proposed a novel methodology for spatial community detection designed to identify clusters of cell types that are frequently colocated. Initially, we defined the cells located within a 30 μm radius of each target cell as its neighbors. We subsequently calculated a proportional vector that represented the cell type composition of these neighboring cells. These vectors served as node representations within a Delaunay triangulation graph. Utilizing a graph neural network model called GraphSAGE^55^, we encoded each cell as a feature vector that captured the local microenvironment as defined by the proportion of its neighboring cells. The model was optimized in an unsupervised manner following the original GraphSAGE training objective^56^ until convergence. Afterward, we applied Leiden clustering to the GraphSAGE-derived features to obtain the spatial “districts” of the cells.

The model was implemented using PyTorch (v1.10), leveraging the PyTorch-geometric implementation of the “SAGEConv” layer (v2.0.3). The SAGEConv layers were used in their default configuration to manage the hidden and output layers, automatically employing root_weight = True, which integrates each cell’s intrinsic feature vector with the aggregated embeddings from its neighbors. Model training was conducted using the Adam optimization algorithm with a learning rate of 0.001 for 150 epochs. The model included a contrastive loss component, configured with a contrastive temperature (tau) of 0.3 and a lambda (lam) of 0.3, further focusing on enhancing the embeddings. The dropout layer, specified with *P* = 0.25.

#### Chi-square test for identifying the migration patterns of plasma cells

We performed a chi-square test to evaluate whether the two migration patterns of plasma cells occurred randomly. Specifically, we counted the number of plasma cells migrating along different axes and calculated the significance of the migration in various directions. Our null hypothesis (H_0_) states that plasma cell migration is random, whereas the alternative hypothesis (H_1_) posits that plasma cell migration is nonrandom.

Next, we calculated the chi-square value (χ²) for the migration of plasma cells from location Di to D_j_ using the following formula:

where O_i_ represents the observed frequency of plasma cells migrating from D_i_ to D_j,_ and E_i_ represents the expected frequency of plasma cells migrating from D_i_ to D_j_. With 19 degrees of freedom (df = 19), we obtained χ² = 30.69 and *P* = 0.0436.

#### Analysis of differentially expressed genes

Differentially expressed genes within each cluster were analyzed using the FindAllMarkers function of the Seurat package (v4.0.6), and the differential expression of genes between the two groups was detected using the FindMarkers function. The parameters were set to min.pct=0.25 and logfc.threshold=0.5.

#### Spatiotemporal analysis of plasma cell movement analysis

The extraction and alignment of the BCR repertoire from enriched CDR3 library sequencing data were conducted using the TRUST4 tool^56^ with the default parameters. Additionally, the IGH clonotype was retrieved from the Stamp-seq snRNA FASTQ file using MiXCR^57^ (v3.0.13), employing the default parameters. The clonotypes of plasma cells from the two sources were combined (Supplementary Table S6). The pseudotime analysis of plasma cells in different districts was performed using Slingshot^58^ (v2.6.0) with the default parameters. Three pseudotime routes were identified (Supplementary Fig. S11b–d). The identified cells with IGH clonotypes were overlapped with *IGHG1*^*+*^ and *IGHA1*^*+*^ plasma cells (507 of 4735), after which plasma cell pairs with the same clonotype and belonging to different districts were isolated. The pair numbers for pseudotime routes 1, 2 and 3 were 44, 4 and 5, respectively. We focused on route 1 because of the apparent abundance of clonal pairs compared with the other two routes. Using the pseudotime for each cell (or the average pseudotime for all cells from the same clonotype in the given district) in each pair, the spatiotemporal movements between these pairs were determined and summed (Fig. 5f).

#### Trajectory analysis

The pseudotime trajectory of epithelial cells was determined with Monocle2^59^ (v2.22.0). The Seurat-derived count matrix was transformed into a CellDataSet format using the importCDS() function. For cell ordering, we identified significantly DEGs (*q* < 0.001) using differentialGeneTest(), which were then specified as ordering genes in setOrderingFilter(). Dimensionality reduction and trajectory modeling were performed using the DDRTree algorithm with reduceDimension(). Temporal gene expression patterns were visualized with plot_genes_in_pseudotime() to monitor transcriptional dynamics over pseudotime.

#### PROGENy analysis

We evaluated the pathway activity of cells from the Stamp-seq snRNA dataset using the PROGENy R package^60^. The “progeny” function from PROGENy (v1.20.0) was executed with the following parameters: scale = FALSE, top = 500, and organism = Human.

#### Scoring gene signatures

HALLMARK gene sets were downloaded from the Molecular Signature Database (MSigDB; https://data.broadinstitute.org/gsea-msigdb/msigdb/release/2024.1.Hs/h.all.v2024.1.Hs.symbols.gmt). Signature scores for malignant cells were calculated using the AddModuleScore function in Seurat. GSEA of HALLMARK pathways between malignant cells in PCR and MPR/nonMPR samples in this study was performed using the GSEA function of the clusterProfiler (v4.6.2) package^61^. Additionally, the GAVA score of apCAFs in the validation dataset (GSE135222 and GSE207422) was calculated using the GSVA (v1.46.0) package^62^.

#### Visualization, quantification, and statistical analysis

The visualization of the spatial distribution of cells was achieved using ggplot2 (v3.4.4). Statistical analyses were conducted using R (version 4.1.1). The survival package (v3.2.13) was used to perform the survival analysis, along with the log-rank test. The pROC (v1.18.0) package^63^ was utilized to calculate the AUC for the apCAF signature, the ratio of *IGHG1* to *IGHA1*, and the combined apCAF signature, along with the ratio of *IGHG1* to *IGHA1* to predict immunotherapy responses in the validation datasets (GSE135222 and GSE207422). Unless stated otherwise, all tests were two-tailed. A P value of less than 0.05 was considered to indicate statistical significance. In all the box plots, the median is indicated by the centerline, while the boxes outline the first and third quartiles.

### Ethical approval

All participants have signed the informed consent. This study adhered to the Declaration of Helsinki and was approved by the Hunan Cancer Hospital Research Ethics Committee (JS001-ISS-CO147).

## Supplementary information

Supplementary Figures

Supplementary Table S1

Supplementary Table S2

Supplementary Table S3

Supplementary Table S4

Supplementary Table S5

Supplementary Table S6

Supplementary Table S7

Supplementary Table S8

Supplementary Table S9

Supplementary Table S10

## Data Availability

The raw sequence data reported in this paper have been deposited in the Genome Sequence Archive^64^ in the National Genomics Data Center^65^, China National Center for Bioinformation/Beijing Institute of Genomics, Chinese Academy of Sciences (GSA-Human: HRA009763 and HRA009766), and are publicly accessible at https://ngdc.cncb.ac.cn/gsa-human.

## Code availability

The code used to process spatial sequencing libraries is available on GitHub (https://github.com/Panyitong/Stamp-seq). A comprehensive overview of the data processing procedures, along with usage instructions, can be accessed at the following link: http://seekspace.seekgene.com/en/v1.0.0/2.tutorial.html.
