# Supplementary material for: Integrating single-nucleus barcoding with spatial transcriptomics via Stamp-seq to reveal immunotherapy response-enhancing functional modules in NSCLC
Source: Cell Discov. 2026 Feb 5;12:10. doi: 10.1038/s41421-025-00861-6 (PMC12877202; doi:10.1038/s41421-025-00861-6)
Supplement: Supplementary file 1 — Supplementary Figures [file 41421_2025_861_MOESM1_ESM.pdf]

Supplementary data for

Integrating Single-Nucleus Barcoding with Spatial Transcriptomics via Stamp-seq to Reveal Immunotherapy Response-Enhancing Functional Modules in NSCLC

Yitong Pan[1,2,6,13], Huan Yan[3,4,13], Jinhuan Han[5,13], Rui Wu[1,2,13], Caiming Xu[12,13], Guang Lei[6,7], Xingyong Ma[5], Ying Guan[5], Zhao Li[8], Junyuan Deng[9], Keyu Li[5], Qingquan Wei[5], Guangxin Zhang[5], Lei Liu[8], Ajay Goel[12,*****], Zhou Yang[10,11,****], Shaozhuo Jiao[5,***], Yongchang Zhang[3,4,**], Chenxi Tian [1,2,14,*]

[1] Computation Biology Department, China National Center for Bioinformation, Beijing 100101, China.

[2] Beijing Institute of Genomics, Chinese Academy of Sciences, Beijing 100101, China.

[3] Early Clinical Trial Center, Hunan Cancer Hospital/The Affiliated Cancer Hospital of Xiangya School of Medicine, Central South University, Changsha, Hunan, 410013, China.

[4] School of Clinical Medicine, Qinghai University, Xining, Qinghai, 810001, China.

[5] SeekGene BioSciences Co. Ltd, Beijing, 102206, China.

[6] Furong Laboratory, Central South University, Changsha, 410078, Hunan, China.

[7] Department of Radiation Oncology, Hunan Cancer Hospital and the Affiliated Cancer Hospital of Xiangya School of Medicine, Central South University, Changsha, Hunan, 410013, China

[8] GeneMind Biosciences Company Limited, Shenzhen, 518001, Guangdong, China.

[9] Department of Endoscopy, Shanghai Tenth Hospital, Tongji University, Shanghai, 200072, China.

[10] Department of Medical Oncology, Shanghai East Hospital, Tongji University School of Medicine, Shanghai, 200070, China.

[11] Department of Oncology, Shanghai Medical College, Fudan University, Shanghai, 200032, China.

[12] Department of Molecular Diagnostics and Experimental Therapeutics, Beckman Research Institute of City of Hope, Biomedical Research Center, Monrovia, CA 91010, USA; City of Hope Comprehensive Cancer Center, Duarte, CA 91010, USA.

[13] These authors contributed equally.

[14] Lead contact

*Co-corresponding authors

File list:

Figure S1-13

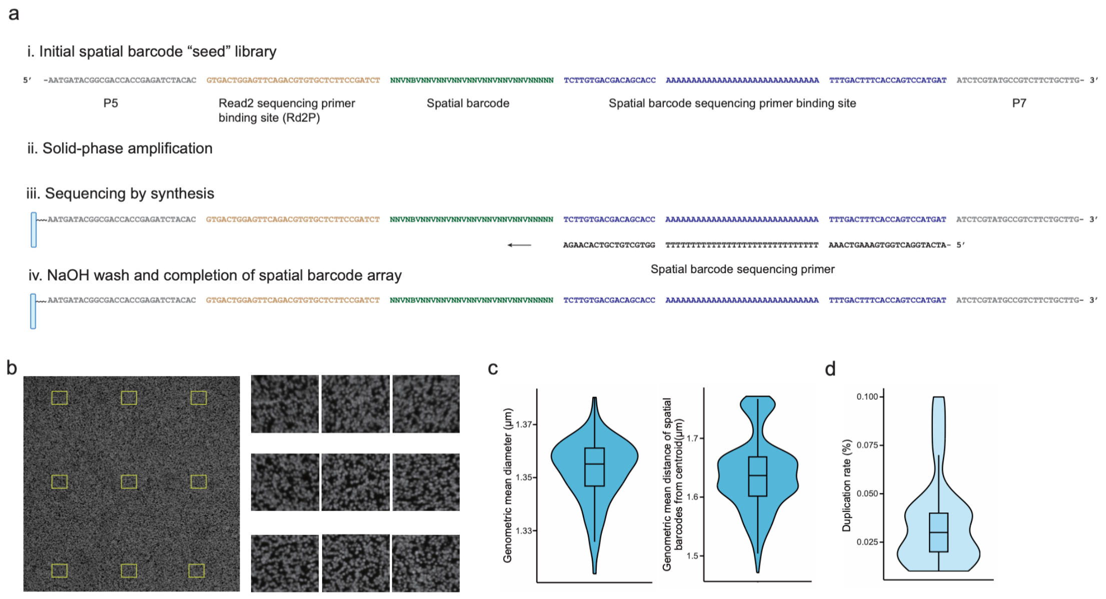

Supplementary Fig. S1. Chip generation procedures and spatial barcode performance characterization (relate to Fig. 1).

(a) Chemistry workflow for chip generation. (b) Representative images of Cycle 33 sequencing results are visualized, showing an overview of oligo clusters (left) and an enlarged view of a selected region (right, yellow square). (c) Box and violin plots illustrate distribution of diameter of spatial fluorescence signal clusters (left) and mean distance between centroids (right). (d) Box and violin plot summarize the duplication rates of spatial barcodes across 29 independently fabricated chips. Boxplots display the median (center line) and upper/lower quartiles (box limits).

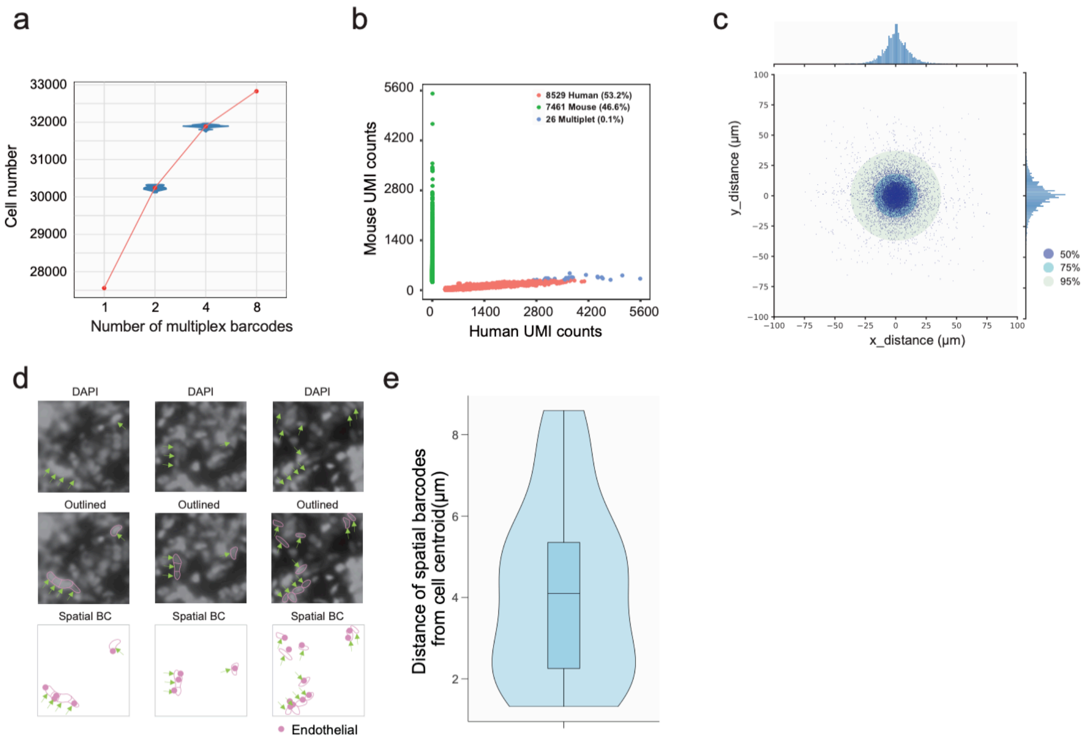

Supplementary Fig. S2. Assessment of Stamp-seq multiplexing efficiency and spatial barcode positioning accuracy.

(a) The line chart displays the mean number of captured nuclei in the WTH1092 Stamp-seq dataset under varying hypothetical numbers of multiplex barcodes. A value of 0 indicates the removal of multiplex barcode information, while a value of 8 indicates the retention of all multiplex barcode information from all nuclei. Violin plots at 2 and 4 multiplex barcodes show the mean number of captured nuclei for random combinations of available barcodes. For example, with two multiplex barcodes, four out of the eight available barcodes are randomly combined and treated as one class. (b) Unique fragments aligning to the human or mouse genomes are quantified. (c) The distance distribution between the spatial coordinates of each localized cell and its corresponding detected spatial barcode is shown. Spatial barcodes located within 25 bins (approximately 0.02 mm^2) around the cell center are included. Points are colored based on two-dimensional kernel density estimation using an axis-aligned bivariate normal kernel, evaluated on a square grid. Kernel density estimates are displayed for both the x and y axes. (d) Stamp-seq-captured cells are overlaid onto a DAPI image in NSCLC samples, demonstrating the alignment of nuclear morphology (green arrows) with cell type annotations (endothelial cells). The upper panel highlights nucleus shapes on the DAPI image. The middle panel outlines the nearest nuclei on the DAPI image, color-coded by cell type based on

Stamp-seq data. The bottom panel shows the corresponding endothelial nuclei locations. (e) Spatial distances between endothelial nucleus position determined by Stamp-seq and their nearest counterparts identified by CellProfiler on the DAPI image are quantified.

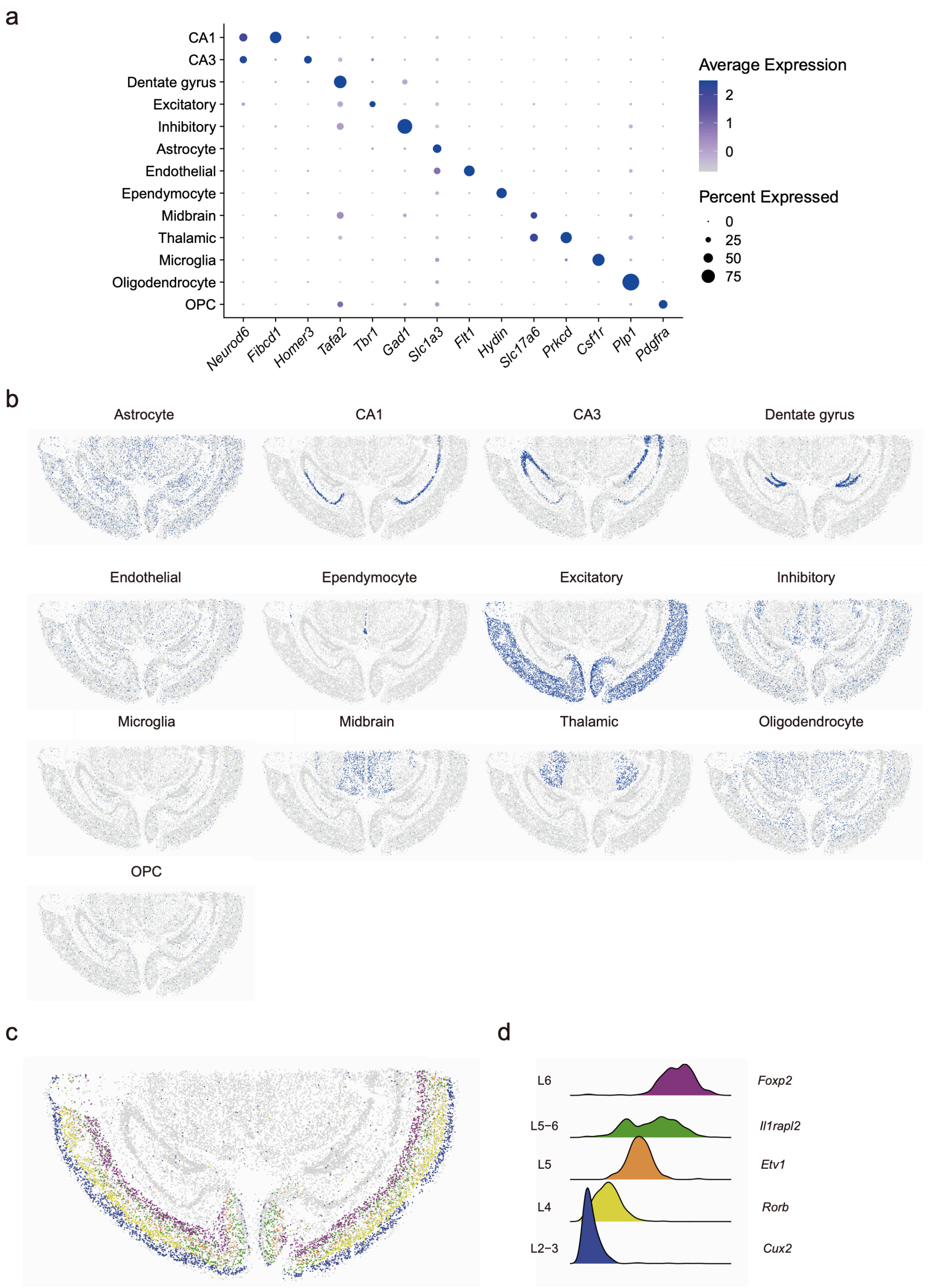

Supplementary Fig. S3. Cell type assignment and spatial mapping in the adult mouse brain.

(a) Dot plot displays the expression levels of marker genes for identified cell type clusters. (b) Spatial distribution of cell type clusters in the mouse brain, annotated as Cornu Ammonis area 1 (CA1), Cornu Ammonis area 3 (CA3), and oligodendrocyte precursor cells (OPC). (c) Spatial distribution of grouped excitatory neuron subtypes across cortical layers 2-6 (L2-6). (d)

Quantification of the spatial distance between each excitatory neuron subtype and the nearest tissue boundary.

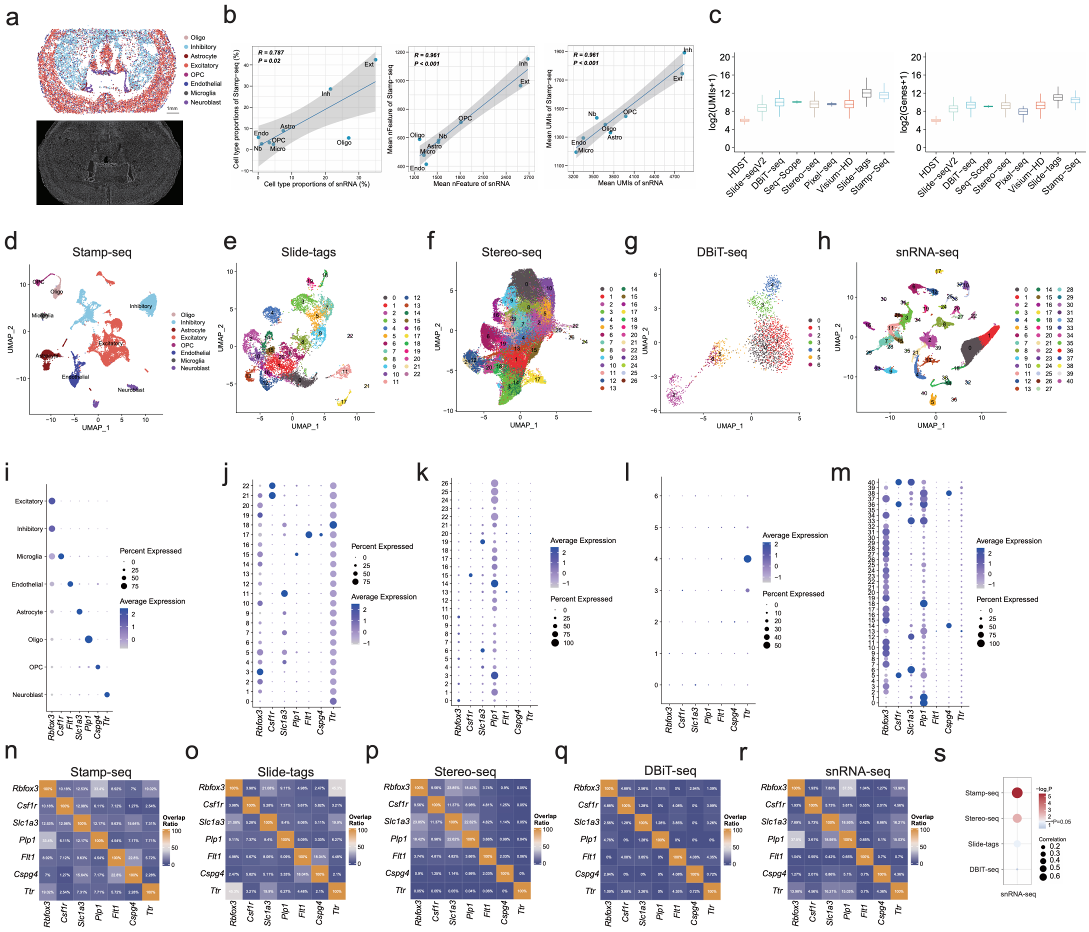

Supplementary Fig. S4. Comparison of Stamp-seq to Slide-tags, Stereo-seq, DBiT-seq, and snRNA-seq.

(a) Stamp-seq enables localization of nuclei to spatial coordinates in the adult mouse brain; cells are colored according to cell type annotation (WTH1059, left) alongside the on-chip DAPI image (right). (b) Comparison metrics plotted against a publicly available adult mouse brain snRNA-seq dataset and Stamp-seq (WTH1059) data. Cell type proportions, mean detected gene counts, and mean UMIs are shown for each cell type. R represents the Spearman correlation coefficient, with shaded area indicating 95% confidence intervals. (c-g) UMAP visualization of snRNA-seq profiles from Stamp-seq (WTH1059), Slide-tags (de novo clusters), snRNA-seq (de novo clusters), as well as 10 µm resolution data from Stereo-seq (de novo clusters) and DBiT-seq (de novo clusters) in the mouse brain. (h-l) Dot plots depicting the expression of selected markers across transcriptome clusters from Stamp-seq, Slide-tags, Stereo-seq, DBiT-seq, and snRNA-seq data in the mouse brain. (m-q) Heatmaps showing the overlap ratio (as a reflection of coexpression) of selected markers (as in h-l) from Stamp-seq, Slide-tags, Stereo-seq, DBiT-seq, and snRNA-seq data in the mouse brain. (r) Dot

plot illustrating the similarity in overlap ratios of selected markers across different platforms, including Stamp-seq, Slide-tags, Stereo-seq, DBiT-seq, and snRNA-seq data. Correlation represents Pearson correlation coefficient.

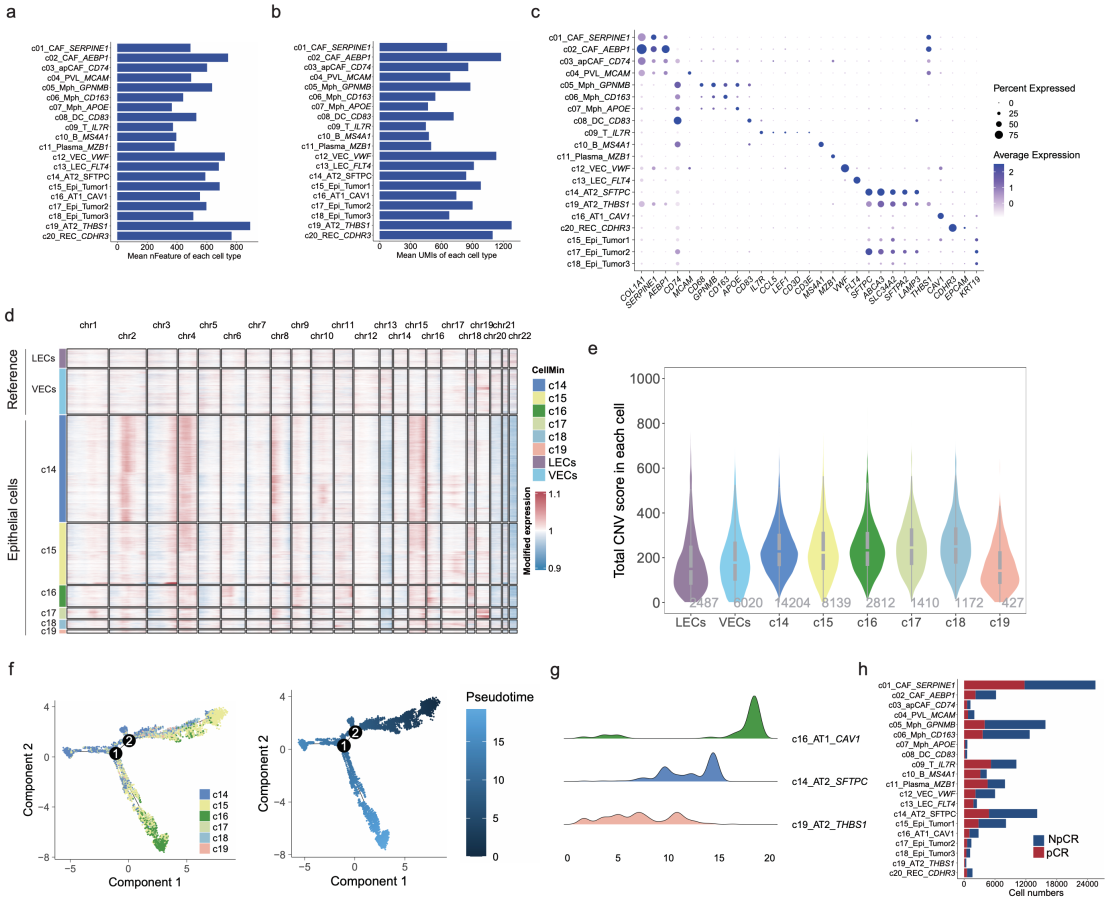

Supplementary Fig. S5. Cell type assignment of NSCLC Stamp-seq data.

(a, b) Bar plots displaying the mean number of genes (a) and UMIs (b) detected per cell subtype. (c) Expression of cell type-specific marker genes across identified subsets. (d) Inferred copy number alterations (CNVs) in epithelial clusters derived from NSCLC Stamp-seq transcriptomic data. (e) Violin plots illustrating the total CNV scores for cells within distinct neoplastic clusters. Grey numbers indicate the cell count in each corresponding cluster. (f) Bar plot depicting the distribution of cell subtypes in pCR and NpCR samples. (g) Ridge plot displaying the pseudotemporal distribution of AT1 and AT2 cells. (h) Bar plot depicting the distribution of cell subtypes in pCR and NpCR samples.

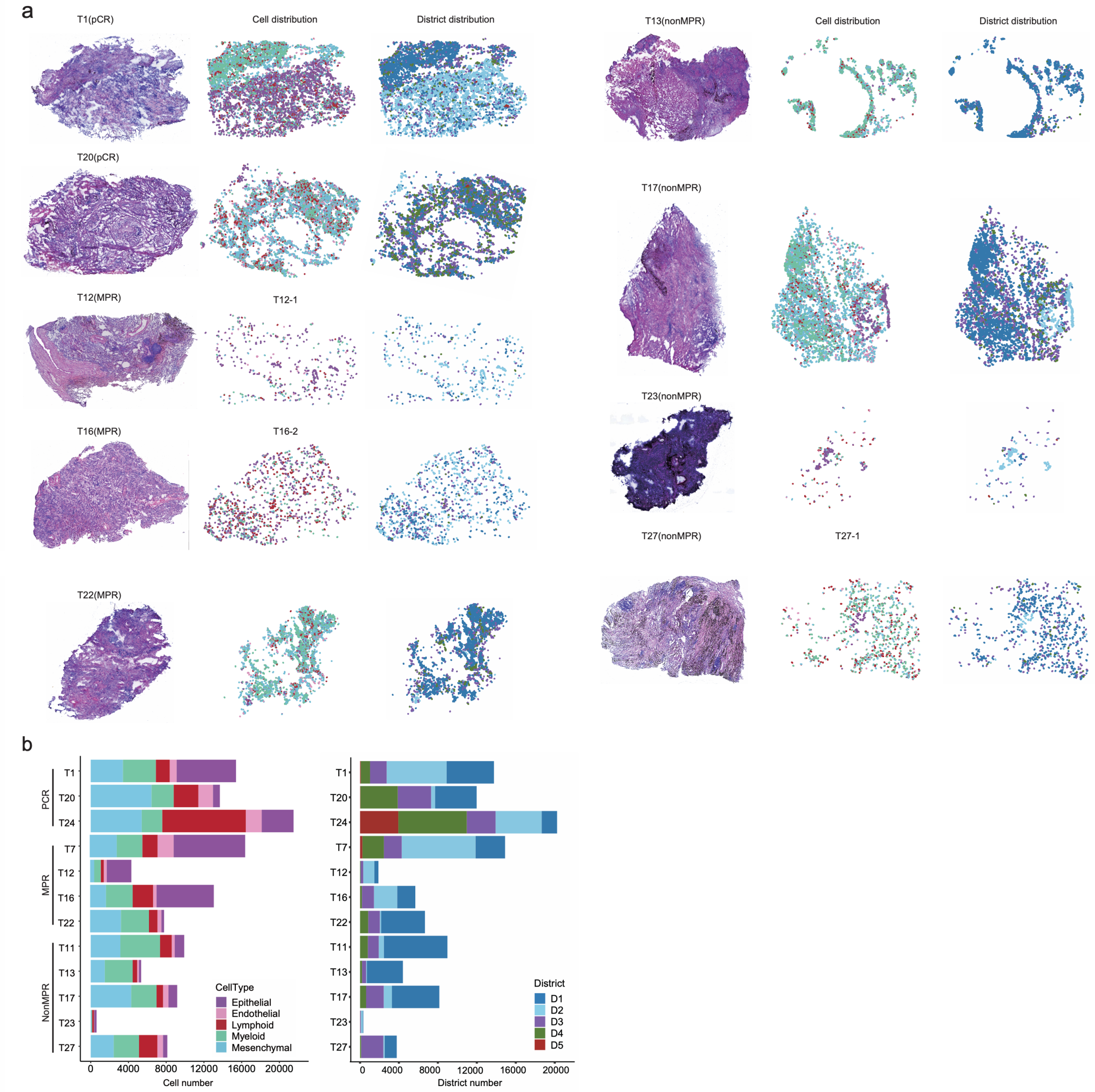

Supplementary Fig. S6. Spatial cell and district distribution in NSCLC samples.

(a) Spatial distribution of distinct cell types (middle) and districts (right) in representative NSCLC samples. (b) Stacked bar plot illustrating the total number of cells (left) and districts (right) in each sample.

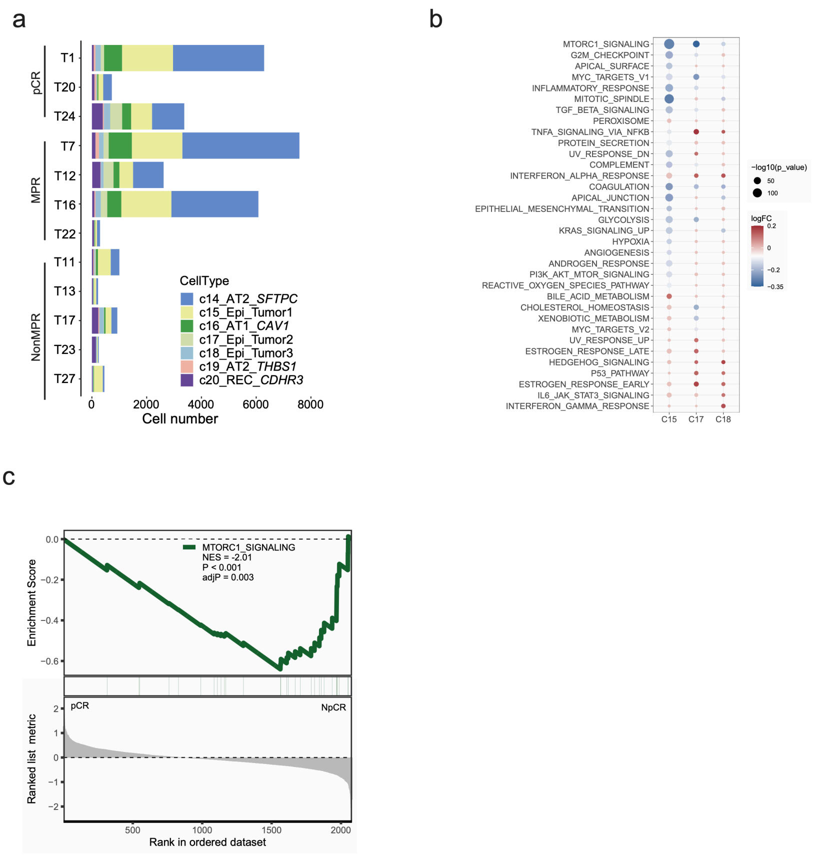

Supplementary Fig. S7. Epithelial cell cluster distribution, copy number alterations and pathway enrichment.

(a) Stacked bar plot depicting the distribution of epithelial cell clusters across different samples. (b) Dot plot illustrating differential HALLMARK pathway activity across three neoplastic cell subtypes. Color represents the $\log_2$ fold change ($\log_2$FC) of pathway scores in pCR compared to NpCR samples, while dot size corresponds to the $-\log_{10}$(P value), with P values calculated using the Wilcoxon test. (c) Gene Set Enrichment Analysis (GSEA) demonstrating the enrichment of proliferation-related pathways in neoplastic cells from NpCR samples compared to pCR samples.

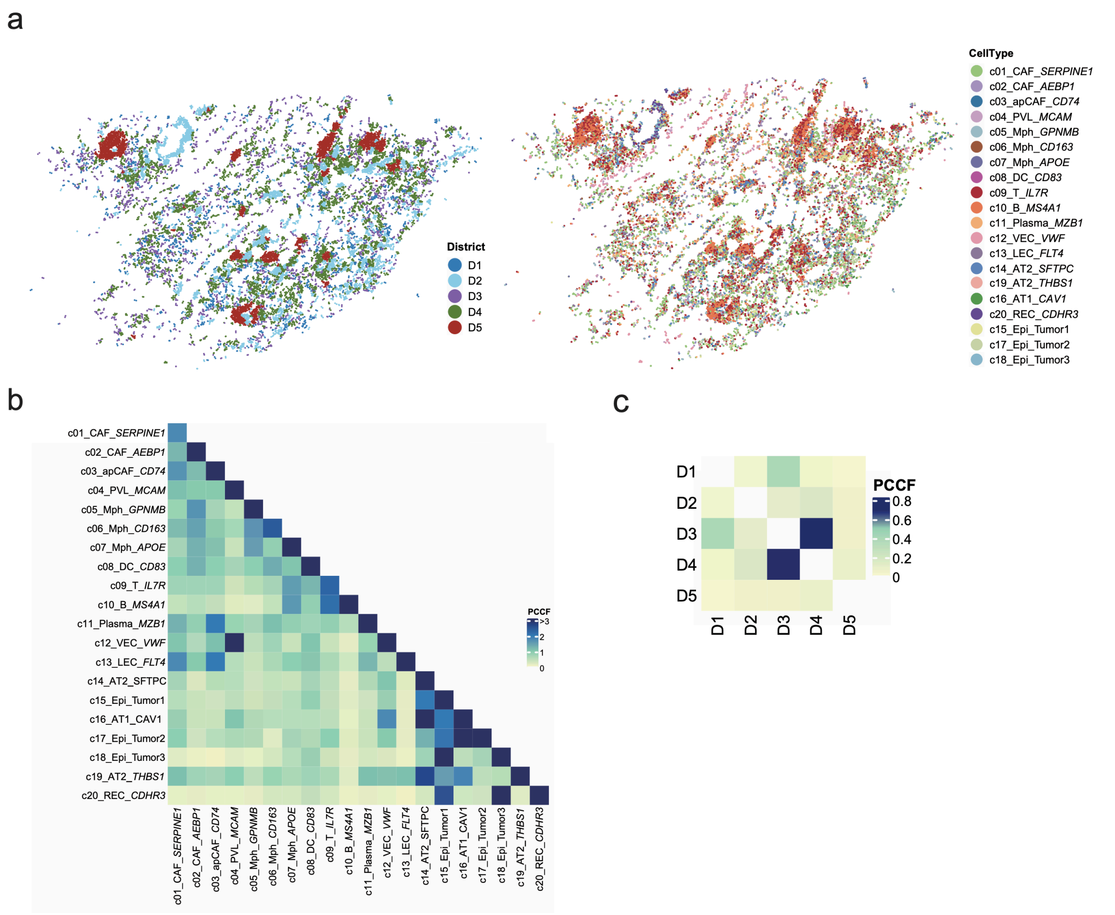

Supplementary Fig. S8. Spatial co-localization and distribution preference of cell types in NSCLC samples.

(a) Spatial mapping of all districts (left) and cell types (right) in the T24 pCR sample. (b) Cell-cell co-localization heatmap demonstrating the degree of spatial co-localization between annotated cell subsets, quantified using Pair Correlation Coefficient (PCCF) statistic scores. (c) District-district co-localization heatmap illustrating spatial co-localization patterns among cells from different districts, with PCCF statistic scores represented on a color scale.

a

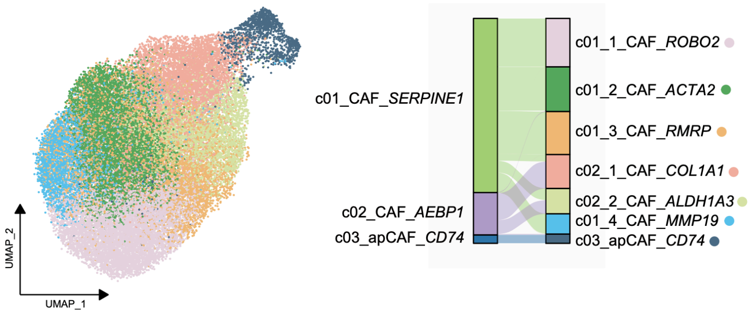

b

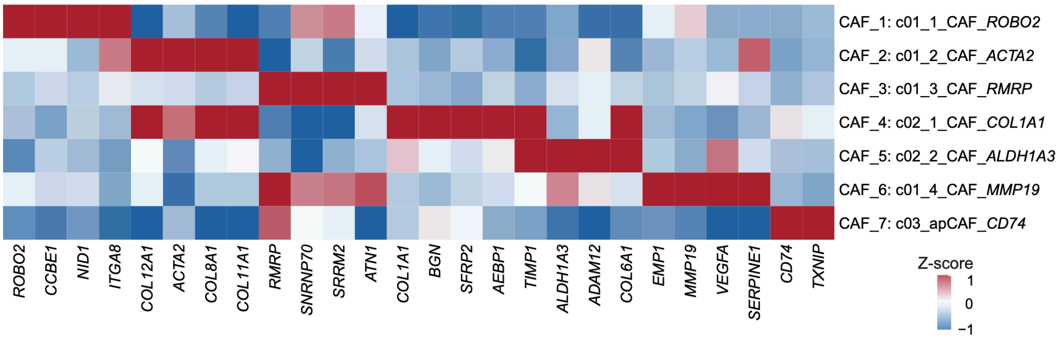

Supplementary Fig. S9. Fibroblast heterogeneity and marker expression in NSCLC.

(a) UMAP plot displaying the distribution of re-clustered fibroblast cell clusters, alongside a stacked bar chart illustrating the correspondence between re-clustered fibroblast subsets and original fibroblast clusters. (b) Heatmap depicting the expression of selected markers across cancer-associated fibroblast (CAF) subtypes.

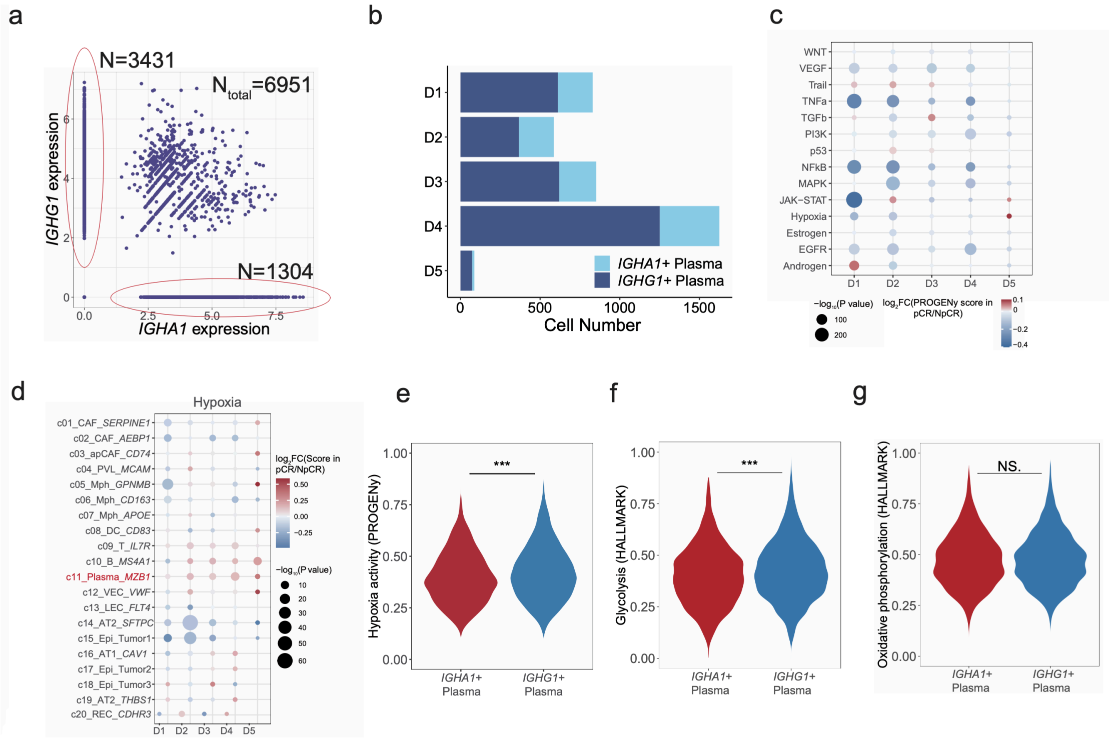

Supplementary Fig. S10. Characterization of plasma cell subtypes and pathway activities across different districts and response groups.

(a) Dot plot showing the expression of *IGHG1* and *IGHA1* genes in plasma cells, categorizing them into *IGHG1*$^+$ and *IGHA1*$^+$ subtypes based on their expression patterns. (b) Quantification of *IGHG1*$^+$ and *IGHA1*$^+$ plasma cells across different districts. (c) Dot plot comparing PROGENy pathway activity across all cells from various districts between pCR and NpCR samples. (d) Dot plot comparing hypoxia pathway activity across all cell types from various districts between pCR and NpCR samples. For panels c and d, color represents the $\log_2$ fold change ($\log_2$FC) of pathway scores between pCR and NpCR samples, while dot size indicates the -$\log_{10}$ (P value), with P values calculated using the Wilcoxon test. (e) Violin plot demonstrating differences in hypoxia pathway activity between *IGHG1*$^+$ and *IGHA1*$^+$ plasma cell populations. (f) Violin plot demonstrating differences in Glycolysis pathway score between *IGHG1*$^+$ and *IGHA1*$^+$ plasma cell populations. (g) Violin plot demonstrating differences in Oxidative phosphorylation pathway score between *IGHG1*$^+$ and *IGHA1*$^+$ plasma cell populations. *** denotes $P < 0.001$ (Wilcoxon test).

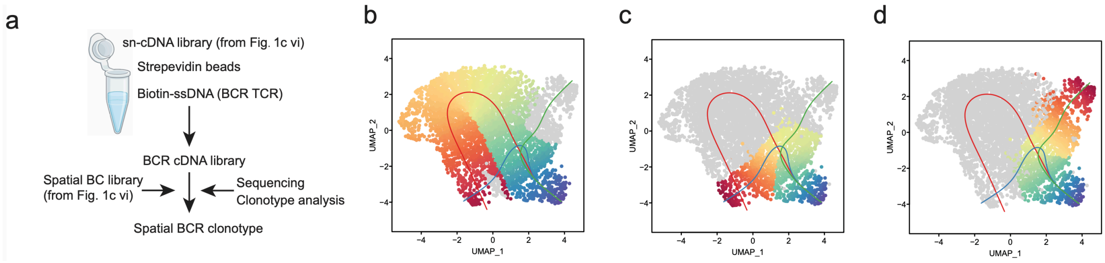

Supplementary Fig. S11. Strategies for spatiotemporal BCR analysis.

(a) Workflow for spatial BCR clonotype analysis. (b-d) Slingshot trajectory analysis illustrating the developmental trajectories of plasma cells, including route 1 (b), route 2 (c), and route 3 (d).

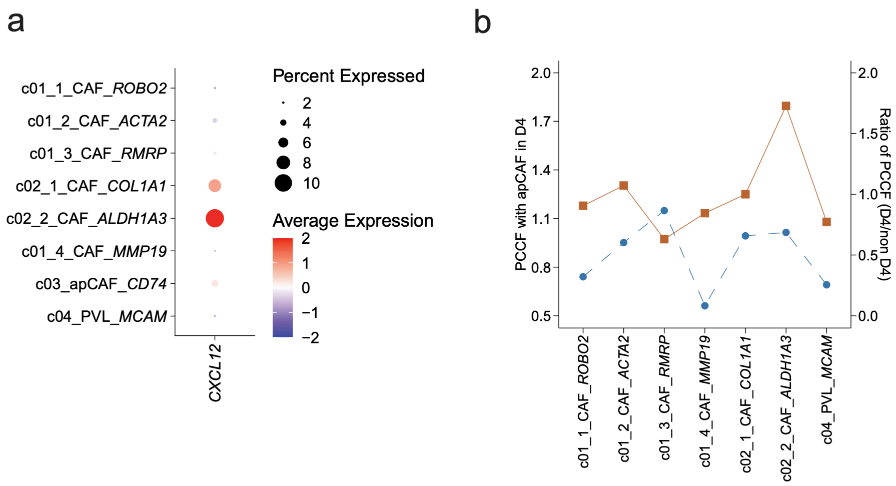

Supplementary Fig. S12. Co-localization of *CXCL12* in CAF subclusters.

(a) Dot plot shows the expression of *CXCL12* in CAF subclusters. (b) The line chart depicts the PCCF value between c03_apCAF_*CD74* and other cell subtypes. The blue points, corresponding to the left Y-axis, represent the PCCF values of c03_apCAF_*CD74* with other cell types specifically within the D4 region. The orange points, corresponding to the right Y-axis, indicate the ratio of the PCCF between c03_apCAF_*CD74* and other cell types in the D4 region versus non-D4 regions (D1, D2, D3, D5).

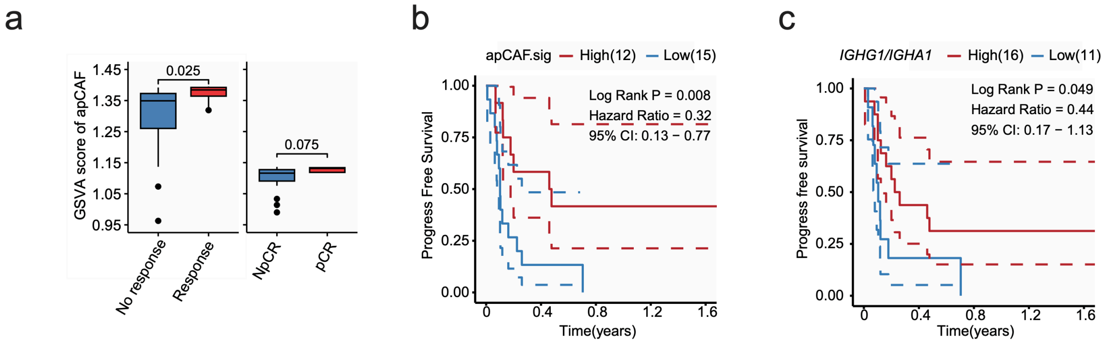

Supplementary Fig. S13. Prognosis values of apCAF and *IGHG1/IGHA1* ratio in NSCLC immunotherapy datasets.

(a) Box plot depicting the GSVA score of apCAF (characterized by *HLA-DRA*, *HLA-DPA1*, *HLA-DQA1*, and *CD74*) between pCR and NpCR samples in GSE135222 (left) and GSE207422 (right) datasets. Wilcoxon P values are shown as numbers above bars. (b, c) Kaplan-Meier estimates of progression-free survival curves for NSCLC patients undergoing immunotherapy, grouped by the GSVA score of apCAF (b) and the *IGHG1*/*IGHA1* ratio (c), in the GSE31245 dataset.
